# Ex vivo study of human visceral nociceptors

**DOI:** 10.1136/gutjnl-2016-311629

**Published:** 2016-09-21

**Authors:** Cian McGuire, George Boundouki, James R F Hockley, David Reed, Vincent Cibert-Goton, Madusha Peiris, Victor Kung, John Broad, Qasim Aziz, Christopher Chan, Shafi Ahmed, Mohamed A Thaha, Gareth J Sanger, L Ashley Blackshaw, Charles H Knowles, David C Bulmer

**Affiliations:** 1 National Centre for Bowel Research and Surgical Innovation, Blizard Institute, Barts and the London School of Medicine and Dentistry, Queen Mary University of London, London, UK; 2 Wingate Institute of Neurogastroenterology, Blizard Institute, Barts and the London School of Medicine and Dentistry, Queen Mary University of London, London, UK

**Keywords:** VISCERAL NOCICEPTION, ABDOMINAL PAIN, ELECTROPHYSIOLOGY, NEUROGASTROENTEROLOGY, VISCERAL SENSITIVITY

## Abstract

**Objective:**

The development of effective visceral analgesics free of deleterious gut-specific side effects is a priority. We aimed to develop a reproducible methodology to study visceral nociception in human tissue that could aid future target identification and drug evaluation.

**Design:**

Electrophysiological (single unit) responses of visceral afferents to mechanical (von Frey hair (VFH) and stretch) and chemical (bradykinin and ATP) stimuli were examined. Thus, serosal afferents (putative nociceptors) were used to investigate the effect of tegaserod, and transient receptor potential channel, vanilloid 4 (TRPV_4_) modulation on mechanical responses.

**Results:**

Two distinct afferent fibre populations, serosal (n=23) and muscular (n=21), were distinguished based on their differences in sensitivity to VFH probing and tissue stretch. Serosal units displayed sensitivity to key algesic mediators, bradykinin (6/14 units tested) and ATP (4/10), consistent with a role as polymodal nociceptors, while muscular afferents are largely insensitive to bradykinin (0/11) and ATP (1/10). Serosal nociceptor mechanosensitivity was attenuated by tegaserod (−20.8±6.9%, n=6, p<0.05), a treatment for IBS, or application of HC067047 (−34.9±10.0%, n=7, p<0.05), a TRPV_4_ antagonist, highlighting the utility of the preparation to examine the mechanistic action of existing drugs or novel analgesics. Repeated application of bradykinin or ATP produced consistent afferent responses following desensitisation to the first application, demonstrating their utility as test stimuli to evaluate analgesic activity.

**Conclusions:**

Functionally distinct subpopulations of human visceral afferents can be demonstrated and could provide a platform technology to further study nociception in human tissue.

**Significance of this study**

**What is already known on this subject?**

- Dysregulation of peripheral afferent sensitivity is an important mechanism in chronic visceral pain; however, current research in this field has been largely limited to animal models.
- Preliminary studies have demonstrated the feasibility of recording from human GI afferents *in vitro*.
- Pilot data suggest that subpopulations of visceral afferents may exist in the human gut.

**What are the new findings?**

- Our studies define the presence of functionally distinct subpopulations of human visceral afferents comparable to those seen in animal studies. Importantly, these include a population of polymodal nociceptors that preferentially respond to algogenic stimuli and are located in the serosa.
- Human serosal visceral nociceptor mechanosensitivity is attenuated by treatment with the transient receptor potential channel, vanilloid 4 (TRPV_4_) antagonist (HC067047), highlighting the therapeutic potential of TRPV_4_ blockade for the treatment of visceral pain.
- Human visceral nociceptor mechanosensitivity is also attenuated by tegaserod suggesting that its efficacy for the treatment of pain in IBS may be mediated through the reduction of visceral nociceptor mechanosensitivity.

**How might it impact on clinical practice in the foreseeable future?**

- Data from our studies will allow a greater understanding of visceral nociceptor function in health and disease.
- The presented methodology provides a platform for the identification of novel therapeutic targets and evaluation of novel putative visceral analgesics.
- There is an approach to basic research on visceral pain that reduces the need for the use of animal experimentation.

## Introduction

Abdominal pain is a common presenting symptom of GI disease. For many patients, this is a chronic problem without any clear underlying pathology. Treating chronic abdominal pain is clinically challenging due to the lack of efficacy or presence of gut-specific side effects of many analgesics. As a result, abdominal pain is a significant factor in the long-term morbidity associated with many GI diseases, impacting negatively on many quality of life indicators such as fatigue, sleep and depression.^1^ ^2^ Conditions characterised by chronic pain are consistently identified as a major burden on the healthcare system^3^ underpinning the need to develop new, safe and effective treatments from both clinical and socioeconomic perspectives.

One approach to analgesic development has been to target sensory nerves called nociceptors that transduce painful or noxious stimuli from the periphery to the central nervous system.^4^ This approach has therapeutic potential as evidenced by the effectiveness of local anaesthetics,^5^ which inhibit peripheral input into the pain pathway. A goal of research in this area has been to identify mechanisms specific to nociceptor activation that can reproduce the efficacy of local anaesthetics without the side effects associated with pan-sodium channel blockade. In particular, the identification of the transient receptor potential (TRP) family of ion channels as key transducers of noxious stimuli such as heat, cold and pressure, and as downstream effectors of receptor activation by inflammatory mediators such as bradykinin, ATP or prostaglandins, has been the focus of substantive investigation.^6^ More recently, the identification of causative loss-of-function or gain-of-function mutations in specific sodium channel subtypes (Na_V_1.7, 1.8, 1.9) selectively expressed in sensory nerves with clinical pain phenotypes supports the concept that targeting nociceptor-specific mechanisms can inhibit pain without affecting other sensory modalities including touch.^7–10^

A limitation to research in this area has been our lack of specific knowledge of *human* nociceptor function leading to reliance on data from model organisms, principally rodent and guinea pig. This is particularly true for visceral nociceptors where current gold standard approaches to studying human nociceptors such as microneurography,^11^ or the use of native/stem cell-derived human sensory neurones are unsuitable.^12^ This is due to the lack of accessibility of visceral nerves, the small proportion of visceral nociceptors within sensory ganglia^13^ and differences in the transduction of noxious stimuli by visceral compared with somatic nociceptors.^14–17^ Surgically resected human bowel can be obtained on a frequent basis from consenting patients undergoing surgery as part of their standard clinical treatment for GI disease. We therefore sought to develop a methodology to study human nociceptor activity ex vivo using surgically resected human bowel.

Visceral nociceptors have been extensively characterised in rodents by their sensitivity to noxious mechanical stimuli (eg, compression of receptive fields with von Frey hair (VFH) filaments, tissue stretch or high pressure distension),^14^ ^18^ ischaemic and hypoxic conditions^19^ and algogenic mediators (eg, bradykinin and ATP).^16^ ^20^ This contrasts with other gut afferent populations that are sensitive to innocuous levels of stretch or light mucosal stroking and which respond to physiological stimuli associated with normal movements of the bowel. Visceral nociceptors have been further characterised in model species, including rodent and guinea pig, into serosal, mesenteric and submucosal subclasses based on the location of their receptive fields in the wall of the gut or mesentery of flat-sheet ex vivo colonic preparations.^14^ Pilot data in human tissue suggest similar subpopulations may exist in man.^21^ ^22^

To identify and characterise nociceptors in human tissue, we assessed electrophysiological responses of discriminated visceral afferent units to the application of mechanical (VFH probing, tissue stretch and mucosal stroking) and chemical (bradykinin and ATP) stimuli to their receptive fields. Further, we examined the effect of tegaserod, a clinically effective treatment of pain in IBS, and transient receptor potential channel, vanilloid 4 (TRPV_4_) antagonism, a high value target for the development of novel visceral analgesics in IBS.^23–25^ Finally, we sought to develop a chemosensitivity paradigm in which the efficacy of other novel analgesics could be tested against noxious inflammatory mediators.

## Methods

All human tissue was collected and used with the approval of the East London and the City HA Local Research Ethics Committee (NREC 10/H0703/71). Resected human ileum, colon, rectum and appendix were collected after written consent from patients undergoing elective surgery as part of their standard clinical treatment at the Barts Health NHS Trust (London, UK). All tissues were obtained from a histopathologist following pathological examination and used either on the day of collection (n=57) or after overnight (12–15 hours) cold storage (4°C) in carbogenated (95% O_2_, 5% CO_2_) Krebs buffer (n=39) (see online supplementary methods).

10.1136/gutjnl-2016-311629.supp1 Supplementary data

### Electrophysiology

**Figure 1:**
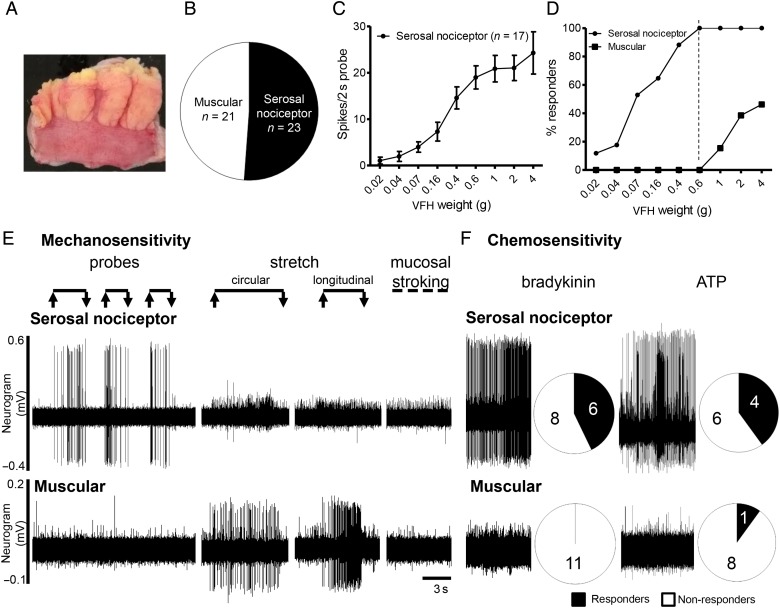
Characterisation of isolated visceral afferent fibres from resected human bowel tissues into serosal nociceptor and muscular afferent subtypes based on responses to differing mechanical and noxious chemical stimuli. (A) Example image of resected bowel tissue pinned flat in the recording chamber. The bowel serosa can be seen below the dissected mesentery. (B) Proportions of muscular and serosal nociceptor subtypes characterised from identified mechanosensitive afferent recordings. (C) Stimulus-response curve to von Frey hair (VFH) probing (0.02–4 g) for serosal afferents in resected human bowel tissues. (D) Associated activation thresholds of VFH probing (0.02–4 g). Dashed line at 0.6 g VFH weight highlights differential activation thresholds of serosal nociceptor (100%) versus muscular subtypes (0%) to VFH probing, potentially allowing subpopulations to be discriminated by VFH probe threshold alone. (E) Example responses to 0.4 g VFH probe, circular and longitudinal tissue stretch and stroking of the gut mucosa in both serosal nociceptor and muscular afferent subtypes. Specifically, serosal nociceptors elicit action potential firing to a range of VFH probe weights tested (0.02–4 g), but are non-responsive to tissue stretch and mucosal stroking. Muscular afferents are responsive to tissue stretch and only respond to VFH probing at weights of >0.6 g. (F) Examples of action potential firing to prototypic algogenic mediators bradykinin and ATP in serosal nociceptor and muscular afferents, and the proportion of responders in each afferent subtype.

Tissues were transferred to a bespoke rectangular recording chamber (100 mm (length)×60 mm (width)×20 mm (depth), with Sylgard base (Dow Corning, UK) and pinned flat with serosal facing up (figure 1A). Tissues were superfused with carbogenated Krebs buffer at a rate of 6 mL/min maintained at 32–34°C supplemented with atropine (10 µM) and nifedipine (10 µM) to prevent smooth muscle contractility. Mesenteric nerve bundles were dissected and recorded using suction electrodes as previously described.^22^

### Experimental protocols

#### Characterisation of visceral nociceptors

Flat-sheet preparations were assessed for mechanosensitivity by serosal probing with a blunt cotton bud, longitudinal and/or circumferential stretch of the tissue with forceps, and in some preparations mucosal stroking with a metal rod. If focal receptive fields were identified, stimulus-response curves were generated using a range of increasing strength VFH probes (0.02, 0.04, 0.07, 0.16, 0.4, 1, 2 and 4 g; 3×3 s probe; at 3 s intervals). The response of mechanosensitive units to the bath application of algogenic mediators bradykinin and/or ATP was then assessed.

#### Effects of existing and novel therapeutic treatments for IBS on visceral nociceptor mechanosensitivity

To confirm the stability of repeated VFH probing, time-matched control experiments were performed. In these experiments, three sets (3×3 s) of VFH probes, separated by 5 min intervals, were performed prior to a 5 min administration of either Krebs buffer or dimethyl sulfoxide (DMSO) (0.1%) and repeated for up to an hour thereafter. A similar protocol was then used to determine the effects TRPV_4_ modulation on visceral nociceptor mechanosensitivity by applying the agonist GSK1016790A (20 mL 10 µM) followed by the antagonist HC067047 (20 mL 100 µM) or in separate studies the 5-hydroxytryptamine (5-HT) receptor 4 partial agonist tegaserod (100 mL 30 µM) (see online supplementary methods).

#### Chemosensitivity of putative nociceptors

In tissues where reproducible mechanosensitivity could not be determined, chemosensitivity to noxious inflammatory mediators was examined. In initial studies, bradykinin, ATP, 5-HT, histamine, prostaglandin E2 (PGE_2_) or capsaicin, were superfused sequentially into the tissue bath to determine the responsiveness of each preparation. Next, the response to repeat applications of bradykinin, ATP, 5-HT, capsaicin or histamine was examined in separate experiments. A small number of preparations were tested for mechanosensitivity before and after the application of bradykinin to test for the recruitment of silent afferents. Finally, specific bradykinin or ATP receptor agonists (B1 receptor agonist Sar-[D-Phe^8^]-des-Arg^9^-Bradykinin, P2X receptor agonist α, β methylene ATP) and antagonists (B1 receptor antagonist R715, B2 receptor antagonist HOE140, adenosine receptor antagonist CGS15943, P2×_2/3_,_3_ receptor antagonist RO4) were used to examine the pharmacology of the responses to these mediators.

### Post hoc analysis

Post hoc analysis were performed to examine the effect of cold storage, age, gender and tissue region, on mechanosensitivity (VFH probing) and chemosensitivity (bradykinin and ATP), in macroscopically normal tissue from surgical resections performed for the treatment of bowel cancer. Additionally, the effects of inflammatory disease were examined by comparing responses with those obtained from inflamed tissue obtained from resections performed for the surgical treatment of IBD, Crohn's disease and UC.

### Data analysis

Data analysis has been described previously.^22^ Neuronal firing rates were examined offline using data analysis software in Spike 2 (CED, UK). Data sets were analysed using the appropriate parametric and non-parametric tests; paired and unpaired t-tests, Fisher's exact tests and Pearson's and Spearman's correlations (see online supplementary methods). Data are expressed as mean±SEM, and statistical significance was set at p<0.05.

### Drugs

Drugs in powder form were solubilised as per the manufacturer's recommendations, aliquoted and frozen at −20°C until required. Aliquots were diluted in Krebs buffer to final working concentrations. Bradykinin, GSK1016790A, capsaicin, 5-HT, histamine, adenosine and ATP were obtained from Sigma-Aldrich (St Louis, Missouri, USA). HC067047, tegaserod, HOE140, R715, CGS15943, α,β-methylene ATP, Sar-[D-Phe^8^]-des-Arg^9^-bradykinin and PGE_2_ were purchased from Tocris Bioscience (Bristol, UK). RO4 was a gift from Neusentis (Cambridge, UK).

## Results

### Characterisation of mechanosensitive units

Forty-six mechanosensitive units were identified in 37/97 pieces of tissue recorded (online supplementary table S1). Of these, two clear subtypes of units could be determined. One subtype found in 23/46 units tested responded to low weight (threshold ≤0.6 g, 100% of units responded at 0.6 g) VFH probing of the serosa but not tissue stretch or mucosal stroking, suggesting localisation within the serosal layer (figure 1B–E). The other subtype found in 21/46 units tested were responsive to either circular or longitudinal tissue stretch (13/18 responding to both) and only responded to high weight VFH probing, if at all (threshold >0.6 g, only 40% of units responded at 4 g max weight tested) suggesting they were located in deeper tissue layers, probably muscle (see figure 1B, D, E and online supplementary tables S2 and S3). These were termed serosal and muscular afferents, respectively, in keeping with nomenclature adopted in animal studies.^14^ One of the 21 muscular units also responded to mucosal stroking fulfilling the criteria for muscular-mucosal units. An additional 2 units had receptive fields in the mesentery fulfilling the criteria for mesenteric units. The mesentery was not routinely probed due to the danger of disturbing the recording electrode positioned in the mesentery.

### Characterisation of mechanosensitive units by response to application of algogenic mediators

**Figure 2:**
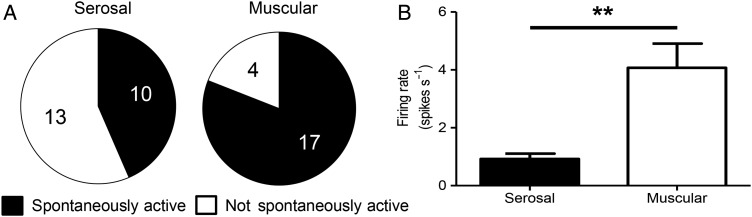
Spontaneous activity in serosal and muscular afferents innervating the human intestine. (A) Pie charts illustrating the proportion of spontaneously active serosal and muscular units. Muscular afferents were significantly more likely to exhibit spontaneous activity (p<0.01, Fisher's exact test). (B) Bar graph demonstrating the firing rate of serosal and muscular units that were spontaneously active. Activity was significantly greater in muscular compared with serosal afferents (**p<0.01, unpaired t-test). Mean±SEM.

The potential role of human serosal and muscular units in nociception was examined by testing their responsiveness to prototypic algogenic mediators, bradykinin and ATP. Robust responses were observed to bradykinin in 6/14 serosal units tested and ATP in 4/10 units tested (figure 1F). By contrast, no muscular unit responded to bradykinin (0/11) and only one muscular unit responded to ATP (1/10), indicating that serosal but not muscular units were likely to function as visceral nociceptors (figure 1F). Consistent with a role as nociceptors the majority of serosal units (13/23) were silent at rest displaying no ongoing discharge. By contrast, a significantly greater proportion of muscular units displayed spontaneous activity (17/21, p<0.05), and this ongoing activity was significantly greater than that displayed by serosal nociceptors (firing rate muscular 4.1±0.8 spikes/s vs serosal 0.9±0.2 spikes/s, p<0.01, figure 2). Mesenteric units also responded to bradykinin and ATP indicative of a role in transducing noxious stimuli.

**Figure 3:**
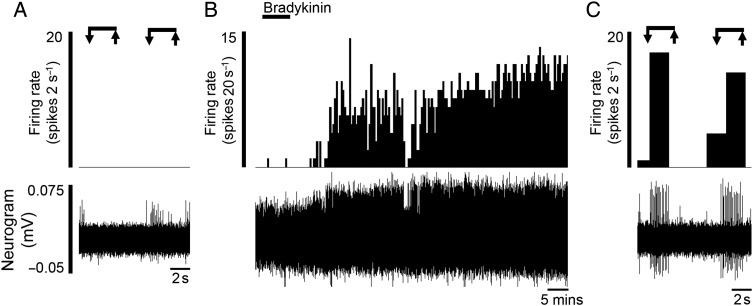
‘Silent’ afferents were evoked after the application of the algogenic mediator bradykinin (n=2). Rate histograms and neurogram showing (A) the lack of response to mechanical probing before bradykinin application, (B) the increase in ongoing activity following application of bradykinin and (C) the acquired mechanosensitivity to von Frey hair probing postbradykinin.

Additionally, we found evidence for the presence of a ‘silent’ nociceptive population that only displayed mechanosensitivity to VFH probing following application of bradykinin (n=2, figure 3 and online supplementary table S4).

### Effect of repeat testing and vehicle

**Figure 4:**
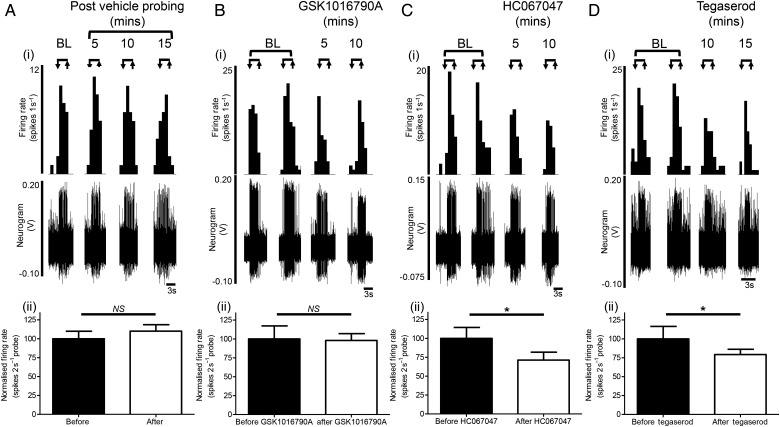
Modulation of mechanosensitive human visceral nociceptors by tegaserod, and the transient receptor potential channel, vanilloid 4 (TRPV_4_) antagonist HC067047. Example rate histogram and neurogram responses of individual von Frey hair probes at baseline (BL) and from the set of probes given within the respective minutes illustrated (eg, 5, 10, 15 min) following (Ai) vehicle (0.1% DMSO/Krebs), (Bi) the TRPV_4_ agonist GSK1016790A, (Ci) the TRPV_4_ antagonist HC067047 or (Di) the partial 5-HT_4_ antagonist tegaserod. Bar graphs illustrating the normalised firing rate per 2 s probe before and after the application of (Aii) vehicle (0.1% DMSO/Krebs) (n=5), (Bii) GSK1016790A (n=6), (Cii) HC067047 (n=7) or (Dii) tegaserod (n=6). Mean±SEM. NS, not significant (p>0.05), *p<0.05, paired t-test.

We examined the stability of serosal nociceptor responses to VFH probing and application of vehicle (0.1% DMSO or Krebs buffer, n=5). Responses to VFH probing were comparable with baseline (100±9.9%), following vehicle application, for example, 5 min (109.6±9.4%, p>0.05), 10 min (114.1±9.4%, p>0.05) and 15 min (106.6±11.5%, p>0.05, figure 4Ai, Aii and online supplementary table S5) postvehicle, and remained stable for a further 30 min afterwards (45 min; 84.9±7.2%, p>0.05), total test period 45 min.

### Effect of TRPV_4_ ligands on visceral nociceptor mechanosensitivity

Compelling data from animal studies demonstrate a role for TRPV_4_ in serosal nociceptor mechanosensitivity,^17^ ^26^ suggesting that TRPV_4_ antagonists could be effective treatments of visceral pain. To investigate this further, we examined the effects of TRPV_4_ ligands on human serosal nociceptor mechanosensitivity. Pretreatment with the TRPV_4_ agonist GSK1016790A had no effect on mechanosensitivity (n=6, figure 4Bi, Bii and online supplementary table S6), but significantly increased baseline activity in 3/8 units tested by ≥50% (see online supplementary figure S1 and table S7). Strikingly, application of the TRPV_4_ antagonist HC067047 (at concentrations that attenuates mouse mechanosensitivity, online supplementary figure S2) significantly reduced human visceral nociceptor mechanosensitivity (−34.9±10.0%, n=7, p<0.05), indicating that TRPV_4_ antagonists may have utility in the treatment of human visceral pain (see figure 4Ci, Cii and supplementary table S8).

### Effect of tegaserod on visceral nociceptor mechanosensitivity

We examined the effects of tegaserod, a clinically effective treatment of abdominal pain in IBS, on serosal nociceptor mechanosensitivity. Tegaserod reduces pain scores in patients with IBS 27 and rectal sensitivity to distension in healthy subjects^28^ indicating an inhibitory effect on pain processing. Animal studies suggest this effect is mediated through a reduction in visceral afferent activity.^29^ Tegaserod significantly reduced human nociceptor mechanosensitivity (−20.8±6.9%, n=6, p<0.05) suggesting that nociceptor inhibition may contribute to the clinical effects of tegaserod IBS (see figure 4Di, Dii and online supplementary table S9).

### Chemosensitivity in putative visceral nociceptors

**Figure 5:**
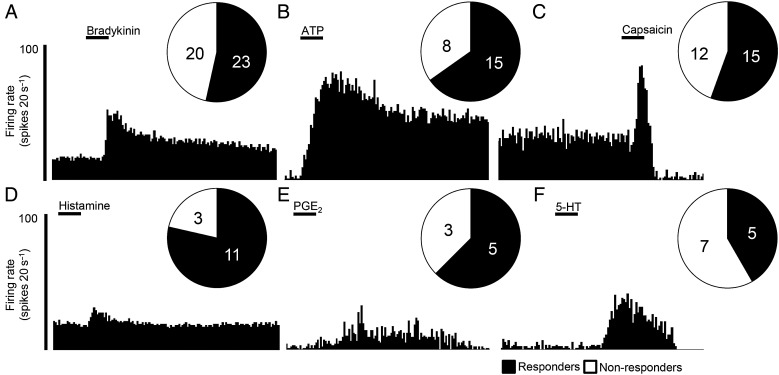
Application of algogenic and disease mediators activates visceral afferents innervating the human intestine. Example of rate histograms illustrating the response profile, and pie charts illustrating the proportion of preparations responding to (A) bradykinin, (B) ATP, (C) capsaicin, (D) histamine, (E) prostaglandin E2 (PGE_2_) and (F) 5-hydroxytryptamine (5-HT).

Finally, chemosensitivity was assessed in the remaining preparations using bradykinin, ATP, capsaicin, 5-HT, histamine and PGE_2_ as prototypic algogenic or disease mediators.^30–33^ At the concentrations tested, the frequency of preparations responding to each mediator ranged from ∼40% to 80% (figure 5). Greater afferent discharge was observed to mediators typically associated with the direct production of pain (bradykinin, ATP and capsaicin) as opposed to mediators associated with nociceptor sensitisation (histamine and PGE_2_) (see figure 5 and online supplementary table S10). In preparations where single units could be clearly discriminated, 8/15 units tested responded to multiple chemical mediators with 4/8 of these units responding to four or more mediators, highlighting the presence of a specific subpopulation of afferents with polymodal sensitivity.

### Repeat administration

**Figure 6:**
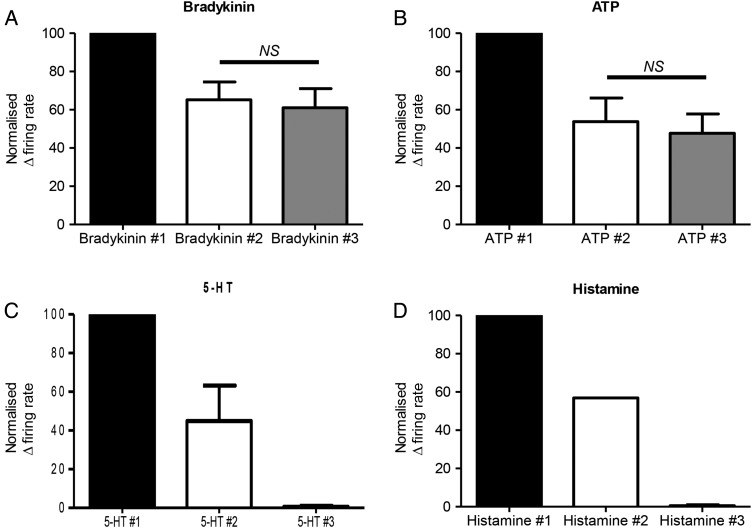
Repeated applications of bradykinin or ATP result in reproducible human afferent responses after initial desensitisation. Bar graphs illustrating the reproducibility of responses to (A) bradykinin (n=6), (B) ATP (n=4), after initial desensitisation to the first application of the respective mediator. A proportion of preparations responded to a second application of (C) 5-hydroxytryptamine (5-HT) (2/2) and (D) histamine (1/2), and no response was seen to a third application of either respective mediator. Mean±SEM. NS, not significant, p>0.05, paired t-test.

To determine which mediators might be suitable for future interventional studies, we examined the effect of repeated mediator applications. The response to the first application of bradykinin or ATP was greater than subsequent applications, as previously reported.^34^ However, after this initial desensitisation, more consistent responses were obtained to the second and third applications of bradykinin (second 65.2±9.3% vs third 61.1±9.9%, of the response to the first application, n=6; online supplementary table S11) or ATP (second 53.8±12.3% vs third 47.7±10.1% of the response to the first application, n=4, p>0.05, figure 6A, B and online supplementary table S12). Responses to a second application of histamine (1/2, online supplementary table S13) or 5-HT (2/2; online supplementary table S14) were greatly reduced. Preparations did not respond to a third application of these mediators (figure 6C, D). Capsaicin (10 μM) produced a marked inhibition of ongoing nerve activity following initial application. Lower concentrations of capsaicin (100 nM) were also tested. This concentration did not inhibit baseline activity, however, only 9 out of 46 units tested responded to two applications of capsaicin, and the second response to capsaicin typically showed marked desensitisation. Responses to PGE_2_ were considered to be of insufficient magnitude to be of utility in an interventional paradigm.

### Mediator pharmacology

**Figure 7:**
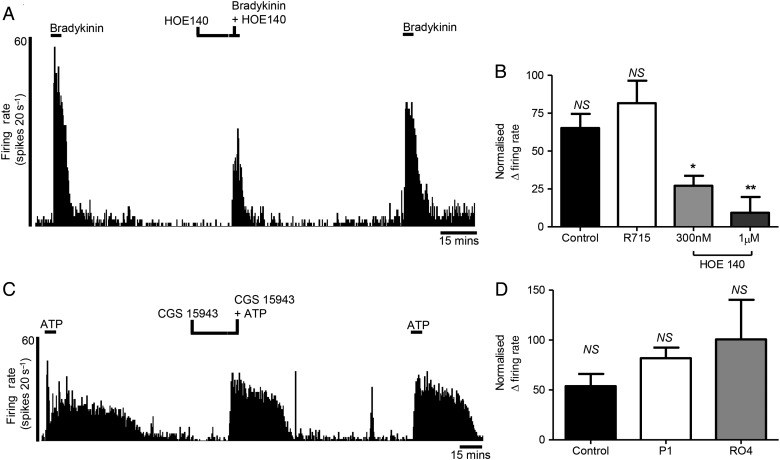
Investigation of receptors involved in the activation of afferents innervating the human intestine by bradykinin and ATP. Example of a rate histogram (A) and bar graph (B) demonstrating the inhibition of human afferent firing in response to bradykinin by pretreatment with the bradykinin receptor 2 antagonist HOE140 (300 nM, n=6, p<0.05; 1 µM, n=4, p<0.01). In contrast, the bradykinin receptor 1 antagonist R715 (n=6) failed to inhibit the human afferent response to bradykinin (B). Example of a rate histogram (C) and bar graph (D) showing the lack of human afferent inhibition in response to ATP when pretreated with the P1 adenosine receptor antagonist CGS15943 (n=6). Similarly, the P2X2/3, 3 receptor antagonist RO4 (n=3) failed to reduce the human afferent response to ATP (D). Mean±SEM. NS, not significant (p>0.05). *p<0.05, **p<0.01, paired t-test.

We explored the pharmacology of the bradykinin and ATP activation of human visceral afferents. Pretreatment with the selective B2 receptor antagonist HOE140 significantly attenuated the afferent response to bradykinin, while treatment with the selective B1 antagonist R715 had no effect (second bradykinin additions: control 65.2±9.3% vs HOE140 300 nM 27.2±6.5%) (n=6, p<0.05; online supplementary table S15) versus HOE140 1 µM 9.3±8.6% (n=4, p<0.05; online supplementary table S16) versus R715 81.6±14.9% (n=6, p>0.05; online supplementary figure S17 and figure 7A,B). The B1 receptor agonist Sar-[D-Phe^8^]-des-Arg^9^-bradykinin had no effect on human afferent activity (0/14 preparations tested, online supplementary figure S3A).

For ATP, pretreatment with the pan-adenosine receptor antagonist CGS15943 or the P2X_2/3_,_3_ antagonist RO4 did not significantly alter the afferent response to ATP (second ATP additions: control 53.8±12.3% vs CGS15943 81.8±10.6%) (n=6, p>0.05, online supplementary table S18) versus RO4 100.7±39.7% (n=3, p>0.05; online supplementary table S19 and figure 7C, D). However, it should be noted that the P2X receptor agonist α,β-methylene ATP can activate human afferents (1/3 preparations tested, online supplementary figure S3C, D).

Additionally, in one unit which displayed stable responses to repeated capsaicin administration, incubation with the transient receptor potential channel vanilloid 1 antagonist ABT-102 abolished the response to a third application of capsaicin. This unit demonstrated washout to a fourth application of capsaicin (see online supplementary figure S4).

### Post hoc analysis

Finally, we performed a post hoc analysis of single unit responses to VFH probing, bradykinin and ATP to confirm the viability of preparations following overnight cold storage; and elucidate differences based on age, gender or inflammatory disease. These differences were examined for different regions of the gut where sufficient data were available.

**Figure 8:**
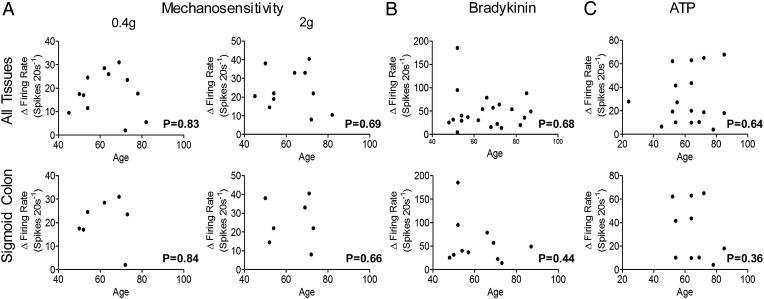
The effect of age on visceral afferent mechanosensitivity and chemosensitivity. Scatter plots illustrating afferent responses to (A) von Frey hair probing at 0.4 and 2 g, (B) bradykinin or (C) ATP compared with the patient's age. Responses were plotted for tissues from cancer resections only, and presented for all tissue regions studied and sigmoid colon only. Pearson's or Spearman's correlations were performed based on data normality.

Afferent responses were comparable following cold storage, with no significant difference seen in the mechanosensitivity or chemosensitivity of stored tissues compared with tissue used immediately (table 1 and online supplementary table S20). Further data analysis did not reveal any significant difference in responses based on gender (table 2 and online supplementary table S21), age (see figure 8 and online supplementary table S20), tissue region (see table 3 and online supplementary table S20) or between normal tissue, and tissues obtained from patients with inflammatory disease (see table 4 and online supplementary table S22).

**Table 1:** The effect of overnight cold storage on visceral afferent mechanosensitivity and chemosensitivity

Mechanosensitivity						
	No storage			Cold storage		
	Firing rate spikes 2 s^−1^ probe (proportion responders)			Firing rate spikes 2 s^−1^ probe (proportion responders)		
VFH (g)	0.07	0.4	2	0.07	0.4	2
All tissues	8.6±2.1 (6/10) (60%)	17.7±2.7 (9/10) (90%)	25.7±3.6 (9/9) (100%)	9.5 (1/3) (33%)	18.3±8.2 (3/3) (100%)	15.0±7.0 (2/2) (100%)
Sigmoid colon	9.0±2.3 (3/5) (60%)	22.3±3.3 (4/5) (80%)	29.6±4.9 (5/5) (100%)	9.5 (1/3) (33%)	18.3±8.2 (3/3) (100%)	15.0±7.0 (2/2) (100%)
Rectum	7.8±7.3 (2/3) (67%)	15.7±6.4 (3/3) (100%)	24.2±5.4 (3/3) (100%)	–	–	–

Bradykinin				
	Proportion responders	Δ Firing rate (spikes 20 s^−1^)	Proportion responders	Δ Firing rate (spikes 20 s^−1^)
All tissues	19/26 (73%)	51.7±10.5	5/13 (39%)	37.7±8.1^NS^
Sigmoid colon	8/13 (62%)	67.3±19.2	4/10 (40%)	32.3±8.4^NS^
Rectum	4/6 (67%)	34.4±18.6	1/1 (100%)	54
ATP				
	Proportion responders	Δ Firing rate (spikes 20s^−1^)	Proportion responders	Δ Firing rate (spikes 20 s^−1^)
All tissues	14/14 (100%)	30.5±8.0	9/12 (75%)	30.2±7.8^NS^
Sigmoid colon	8/8 (100%)	32.4±10.9	6/8 (75%)	33.2±12.7^NS^
Rectum	3/3 (100%)	31.3±18.6	1/1 (100%)	20

Table illustrating single unit responses to VFH probing at 0.07, 0.4 and 2 g, bradykinin or ATP in tissues used on the day of operation or following cold storage. The data were analysed for tissue from cancer resections only, and presented for all tissue regions studied, sigmoid colon only and rectum only. Mean±SEM; p>0.05; paired t-test.

NS, not significant; VFH, von Frey hair.

**Table 2:** The effect of gender on visceral afferent mechanosensitivity and chemosensitivity

Mechanosensitivity						
	Male			Female		
	Firing rate spikes 2 s^−1^ probe (proportion responders)			Firing rate spikes 2 s^−1^ probe (proportion responders)		
VFH (g)	0.07	0.4	2	0.07	0.4	2
All tissues	11.8±1.4 (4/9) (44%)	18.1±3.4 (8/9) (89%)	21.5±4.5 (7/7) (100%)	4.7±2.1 (3/4) (75%)	17.4±4.8^NS^ (4/4) (100%)	27.6±4.7^NS^ (4/4) (100%)
Sigmoid colon	11.5±2.0 (2/6) (33%)	19.1±4.7 (5/6) (83%)	21.4±5.4 (5/5) (100%)	6.8±0.3 (2/2) (100%)	24.3±6.8 (2/2) (100%)	35.5±2.5 (2/2) (100%)
Rectum	15 (1/1) (100%)	26 (1/1) (100%)	33 (1/1) (100%)	0.5 (1/2) (50%)	10.5±1.0 (2/2) (100%)	19.8±0.8 (2/2) (100%)

Bradykinin				
	Proportion responders	Δ Firing rate (spikes 20 s^−1^)	Proportion responders	Δ Firing rate (spikes 20 s^−1^)
All tissues	15/23 (65%)	58.5±12.0	9/16 (56%)	30.2±5.9*^NS^*
Sigmoid colon	8/15 (53%)	69.6±22.1	4/8 (50%)	36.9±7.3*^NS^*
Rectum	3/3 (100%)	52.6±20.9	2/4 (50%)	16.9±12.3
ATP				
	Proportion responders	Δ Firing rate (spikes 20 s^−1^)	Proportion responders	Δ Firing rate (spikes 20 s^−1^)
All tissues	17/18 (94%)	35.4±6.4	6/8 (75%)	13.8±3.2*^NS^*
Sigmoid colon	12/13 (92%)	35.3±8.4	2/3 (67%)	10.4
Rectum	2/2 (100%)	43.9±23.9	2/2 (100%)	13.0±6.4

Table illustrating single unit responses to VFH probing at 0.07, 0.4 and 2 g, bradykinin or ATP in tissues from male or female patients. The data were analysed for tissue from cancer resections only, and presented for all tissue regions studied, sigmoid colon only and rectum only. Mean±SEM; p>0.05; paired t-test.

NS, not significant; VFH, von Frey hair.

**Table 3:** The effect of tissue region on visceral afferent mechanosensitivity and chemosensitivity

	All tissues	Sigmoid colon	Rectum
**Mechanosensitivity VFH (g)**	Firing rate spikes 2 s^−1^ probe Proportion responders	Firing rate spikes 2 s^−1^ probe Proportion responders	Firing rate spikes 2 s^−1^ probe Proportion responders
0.07	8.8±1.8 7/13 (54%)	9.1±1.6 4/8 (50%)	7.8±7.3 2/3 (67%)
0.4	17.8±2.7 12/13 (92%)	20.6±3.7 7/8 (88%)	15.7±5.2 3/3 (100%)
2	23.7±3.3 11/11 (100%)	25.4±4.6 7/7 (100%)	24.2±4.4 3/3 (100%)
** Chemosensitivity**	Δ Firing rate spikes 20 s^−1^ Proportion responders	Δ Firing rate spikes 20 s^−1^ Proportion responders	Δ Firing rate spikes 20 s^−1^ Proportion responders
Bradykinin	49.1±8.6 24/39 (62%)	57.7±14.7 12/23 (52%)	38.3±14.9*^NS^* 5/7 (71%)
ATP	30.3±5.4 23/26 (88%)	32.8±7.9 14/16 (88%)	28.5±13.5*^NS^* 4/4 (100%)

Table illustrating single unit responses to VFH probing at 0.07, 0.4 and 2 g, bradykinin or ATP in tissues from different tissue regions. The data were analysed for tissue from cancer resections only. Mean±SEM;p>0.05; paired t-test.

NS, not significant; VFH, von Frey hair.

**Table 4:** The effect of having IBD on visceral afferent mechanosensitivity and chemosensitivity. Table illustrating single unit responses to VFH probing at 0.07, 0.4, and 2 g, bradykinin, or ATP in tissues from patients having surgical resections for cancer, and patients having resections for IBD

Mechanosensitivity						
	Cancer			IBD		
	Firing rate spikes 2 s^−1^ probe (proportion responders)			Firing rate spikes 2 s^−1^ probe (proportion responders)		
VFH (g)	0.07	0.4	2	0.07	0.4	2
All tissues	8.8±1.8 (7/13) (54%)	17.8±2.7 (12/13) (92%)	23.7±3.3 (11/11) (100%)	6.5±0.5 (2/2) (100%)	11.0±0.0 (2/2) (100%)	13.8±2.8 (2/2) (100%)
Sigmoid colon	9.1±1.6 (4/8) (50%)	20.6±3.7 (7/8) (88%)	25.4±4.6 (7/7) (100%)	–	–	–
Rectum	7.8±7.3 (2/3) (67%)	15.7±5.2 (3/3) (100%)	24.2±4.4 (3/3) (100%)	7 (1/1) (100%)	11 (1/1) (100%)	11 (1/1) (100%)

Bradykinin				
	Proportion responders	Δ Firing rate (spikes 20 s^−1^)	Proportion responders	Δ Firing rate (spikes 20 s^−1^)
All tissues	24/39 (62%)	49.1±8.6*^NS^*	7/10 (70%)	23.5±6.9*^NS^*
Sigmoid colon	12/23 (52%)	57.7±14.7	1/1 (100%)	52.5
Rectum	5/7 (71%)	38.3±14.9	2/3 (67%)	22.6±6.4
ATP				
	Proportion responders	Δ Firing rate (spikes 20 s^−1^)	Proportion responders	Δ Firing rate (spikes 20 s^−1^)
All tissues	23/26 (88%)	30.3±5.4	2/4 (50%)	29
Sigmoid colon	14/16 (88%)	32.8±7.9	1/1 (100%)	–
Rectum	4/4 (100%)	28.5±13.5	0/1 (0%)	–

Table illustrating single unit responses to VFH probing at 0.07, 0.4 and 2 g, bradykinin or ATP in tissues from patients having surgical resections for cancer, and patients having resections for IBD. The data were presented for all tissue regions studied, sigmoid colon only and rectum only. Mean±SEM; p>0.05; paired t-test.

NS, not significant; VFH, von Frey hair.

## Discussion

We present a comprehensive investigation, demonstrating the feasibility of studying human nerve endings in situ. Our approach measures action potential firing, the propagating signal in nociceptors, as opposed to other surrogate markers of nerve activation (calcium fluxes or transmitter release). Additionally, we retain the structural complexity of the nerve terminal architecture in conjunction with local cellular interactions, thereby providing an extension and complementary approach to existing cell-based methodologies such as use of isolated human dorsal root ganglia neurons.^35^ ^36^

We have characterised two functional subtypes of human visceral afferent fibres innervating the bowel in detail. These are readily distinguished in a manner analogous to widely used classifications in rodent nerves.^14^ Specifically, muscular and serosal subtypes defined by the sensitivity of the former but not the latter to tissue stretch, and a clear separation in the activation threshold to VFH probing between the two subtypes. For example, serosal units have low intensity VFH thresholds consistent with a superficial location of their receptive fields in the serosa, while muscular units have a higher threshold suggestive of a receptive field located deeper in the muscle layers. We also provide evidence for other functional subtypes, namely mesenteric, silent nociceptors and muscular-mucosal afferents suggesting that the modality of sensory signalling from the bowel is functionally conserved from rodent to human. In addition, serosal but not muscular afferents were responsive to algogenic mediators, and possessed a paucity of spontaneous activity suggesting that serosal units were most likely nociceptors, and hence transmit pain from the viscera in response to tissue damaging stimuli. By contrast, muscular units, being responsive to low threshold stretch of the bowel, are more likely to transduce physiological stimulation, for example, the passage of bolus through the gut.

We evaluated the effect of existing and potential therapeutic treatments for visceral pain on mechanosensitivity to VFH probing. In keeping with the rodent literature, the application of TRPV_4_ agonists or antagonists enhanced or inhibited human visceral nociceptor activity, respectively.^17^ Combined with studies showing endogenous lipid mediators, which stimulate TRPV_4_, are elevated in IBS,^25^ our data suggest that TRPV_4_ antagonists could be clinically effective in IBS. Further studies are needed to confirm these initial findings, particularly using TRPV_4_ ligands from alternative chemotypes^17^ ^26^ to support selectivity of action over other TRP family members.^37^ ^38^ We also demonstrated an inhibitory effect of tegaserod on human visceral nociceptor mechanosensitivity illustrating how our studies provide information on the reverse translation of clinically effective drugs, in addition to supporting rodent data suggesting a peripheral site of action of tegaserod.

Chemosensitivity was assessed to a range of noxious, inflammatory mediators in preparations in which defined mechanosensitive units could not be identified. We chose to examine responses to capsaicin and bradykinin as prototypic noxious mediators that elicit pain following local injection in humans.^19^ ^20^ ATP was examined, due its release from the viscera following inflammation or distension and algesic effects following dermal injection in humans,^30^ ^31^ while 5-HT, PGE_2_ and histamine were selected due to their increased production in the bowel of patients with IBS^32^ and clinical efficacy of agents modulating their pharmacology.^39^ The goal of these studies was to evaluate the effect of each mediator on visceral afferent signalling, and develop an alternative experimental protocol which could be used to investigate the analgesic potential of novel therapeutic approaches. We showed a subpopulation of fibres are sensitive to a range of mediators, indicating that drugs which block convergent points in the activation of nociceptors by multiple algogenic mediators are needed.^4^ We also examined the stability of repeated mediator application, highlighting the suitability of bradykinin and ATP for use as test stimuli. Afferent responses to capsaicin, histamine and 5-HT showed progressive desensitisation to repeat application, and capsaicin also inhibited ongoing nerve activity. The inhibitory effect of capsaicin is consistent with its clinical use in topical creams, which treat pain by desensitising nociceptors.^40^ By contrast, the pathophysiological implications of desensitising responses to 5-HT and histamine is unclear given the clinical efficacy of 5-HT_3_ antagonists and histamine H1-receptor antagonists in IBS, and may be a feature of this experimental system.^41^ ^42^

Further investigation confirmed that bradykinin stimulates human visceral afferent activity via B2 receptors consistent with rodent data.^15^ ^43^ While responses to repeated application of ATP were not sensitive to blockade with a selective P2X_2/3_ antagonist (RO4) or pan-P1 adenosine receptor antagonist (CGS15493), suggesting the response to ATP in our experimental paradigm was largely driven by P2Y receptor activation, consistent with recent findings in mouse and human tissue.^44^ Importantly, our work does not preclude the activation of human visceral afferents by P2X_2/3_ or P1 receptors. We have found excitatory responses to the P2X_2/3_ agonist, α,β-methylene ATP (see online supplementary figure S3B–D), highlighting the redundancy and complexity of purinergic signalling.

Post hoc analysis of mechanosensitivity and chemosensitivity demonstrated stability to overnight cold storage of tissue, suggesting that tissue could be transported over distance to recipient laboratories. Additionally, no significant differences were seen in responses analysed by age, gender or tissue region, suggesting that the function of individual fibres may not be greatly affected by these variables. This does not exclude the possibility that differences exist. For example, the tissue region used may be important as the anatomical innervation by vagal, splanchnic and pelvic afferent nerves is markedly different between regions of the gut and functional responses to different stimuli can vary.^45^ However, we applied specific stimuli that preferentially targeted spinal nociceptors consistent with a lack of regional differences in responses. In addition, the recent demonstration of reduced visceral afferent sensitivity to noxious stimuli in aged mice,^46^ and reduced afferent activity with age in human tissue,^47^ ^48^ highlights the need for further studies on visceral nociception in human tissue.

A further consideration with the use of human tissue is the diversity of patients' backgrounds. This will result in greater variability of responses compared with tissue from experimental animals, making small changes difficult to see without large sample numbers. Our tissue is obtained from patients with disease, which may polarise patient demographics compared with the populace as a whole. This is particularly apparent in the age and gender of the patients, for example, the majority of our normal tissues came from male patients aged between 50 and 70 years, and our female tissues comes from postmenopausal women.

Finally, our findings in diseased human tissue (IBD) demonstrate how this approach will provide insight into the pathophysiology of naturally occurring disease. Our data set does not support a marked change in sensitivity of visceral afferents in IBD, although clearly more work is needed to understand if this is related to disease or an effect of patient treatments, for example, the use of steroids or antitumour necrosis factor antibodies which could have an effect on afferent signalling.^49^ ^50^

The use of human visceral nociceptor recordings has great future potential enabling further investigation of the physiology of human nociceptor stimulus transduction, the pharmacology of nociceptor signalling and as a translational platform for the validation and identification of future novel visceral analgesics. As highlighted, a pragmatic approach needs to be taken when using human tissue, particularly when interpreting negative findings; however, there is clear value in using human tissue to study specific, well-designed, research questions on human visceral nociceptor function.
