# Supplementary material for: Ex vivo study of human visceral nociceptors
Source: Gut. 2016 Sep 21;67(1):86–96. doi: 10.1136/gutjnl-2016-311629 (PMC5754853; doi:10.1136/gutjnl-2016-311629)

EX VIVO STUDY OF HUMAN VISCERAL NOCICEPTORS

Cian McGuire[1], George Boundouki[1], James R F Hockley[1,2], David Reed[1,2], Vincent Cibert-Goton[1], Madusha Peiris[2], Victor Kung[1], John Broad[1], Qasim Aziz[2], Christopher Chan[1], Shafi Ahmed[1], Mohamed A Thaha[1], Gareth J Sanger[1], L Ashley Blackshaw[1,2], Charles H Knowles[1], David C Bulmer[1,2]

[1] National Centre for Bowel research and Surgical Innovation, Blizard Institute, Barts and the London School of Medine and Dentistry, Queen Mary, University of London, London, UK

[2] Wingate Institute of Neurogastroenterology, Blizard Institute, Barts and the London School of Medine and Dentistry, Queen Mary, University of London, London, UK

CORRESPONDING AUTHOR

Dr David Bulmer

Wingate Institute of Neurogastroenterology

26 Ashfield Street

London E1 2AJ

United Kingdom

Email: d.bulmer@qmul.ac.uk; Tel: +44 207 882 2639

KEYWORDS

Visceral pain, human, translation, nociception,

ABBREVIATIONS

5-HT: 5-hydroxytryptamine; ATP: Adenosine trisphosphate; DMSO: Dimethyl sulfoxide; IBD: Inflammatory bowel disease; IBS: Irritable bowel syndrome; PGE2: Prostaglandin E_2; TRPV: Transient receptor potential vanilloid; VFH: von Frey hair.

WORDCOUNT

2294

SUPPLEMENTARY METHODS

Patients and surgical procedure

All human tissue was collected and used with the approval of the East London and the City HA Local Research Ethics Committee (NREC 10/H0703/71). Resected human ileum, colon, rectum and appendix were collected after written consent from patients undergoing elective surgery as part of their standard clinical treatment at the Barts Health NHS Trust (London, UK). All tissues were obtained from a histopathologist following clinical examination. Macroscopically normal tissues were obtained from patients with non-obstructive tumours (n=60) (a minimum of 10cm from the tumour or lymphatic drainage field) and non-pathological tissue from patients with diverticular disease (n=8), polyps (n=3), stoma reformation (n=2), chronic constipation (n=2), persistent prolapse (n=1), primary sclerosing cholangitis (n=1), adhesions (n=1), stricture (n=1), or trauma (n=1); collectively these were deemed as 'non-inflamed tissue'. Tissues collected from patients with ulcerative colitis (n=7), Crohn's disease (n=8) appendicitis (n=2) were considered as 'inflamed tissues'. The regional distribution of tissues was ileum (n=11), appendix (n=8), colon (n=67) and rectum (n=11) (table 1). In some cases (n=40) tissues were stored overnight (12-15hrs) in a sealed 1L flask containing carbogenated (95% O_2, 5% CO_2) Kreb's buffer (in mM: 124.0 NaCl, 4.8 KCl, 1.3 NaH_2PO_4, 1.2 $MgSO_4.7H_20$, 2.5 $CaCl_2$, 11.1 Glucose, 25.0 $NaHCO_3$) at 4°C and electrophysiological experimentation performed the following day. Alternatively tissues were used immediately after collection (n=57).

Electrophysiology

Tissues obtained from the histopathologist were washed with Kreb's buffer to remove faecal matter. Gross dissection of appendix, ileum, colon and rectum surgical samples was performed to remove tissues suitable for electrophysiological recordings. Specifically appendices and associated mesentery were cut away at the base of the caecum; bowel specimens were opened on the anti-mesenteric border and an approximately square (3cm x 3cm) full-thickness segment removed encompassing a portion of mesenteric attachment (figure 1A). Segments were selected based on the presence of blood vessel arcades within the mesentery with which visceral nerves are known to strongly associate[1]. The tissue was transferred to a bespoke rectangular recording chamber 100mm (length) x 60mm (width) x 20mm (depth), the base of which had been coated in Sylgard (Dow Corning, UK) and pinned flat (appendix kept intact and pinned) with the serosal side facing up. The mesenteric attachment of the tissues was pinned out flat above the bowel wall itself (figure 1A). Tissues were superfused with carbogenated Kreb's buffer at a rate of 6ml/min maintained at 32-34 ^oC via an inline heater; this was supplemented with atropine (10µM) and nifedipine (10µM) to

prevent smooth muscle contractility. Using sharp dissection forceps and a stereomicroscope (SZ40, Olympus, UK) nerve bundles were identified within the mesenteric attachment and the surrounding connective tissue was carefully dissected away. Isolated nerve bundles were recorded from using borosilicate glass electrodes (Harvard Apparatus, UK) backfilled with Kreb's buffer. Neuronal activity was recorded with reference to a common silver wire reference/ground electrode using a differential amplifier (gain 5K) and headstage (Neurolog Ltd, UK). The resultant signal was band pass (100-1500 Hz; Neurolog, UK) and digitally filtered (50 Hz; Humbug, Quest Scientific, UK), before being digitised (20kHz, 1401 micro Cambridge Electronic Design; CED, UK) and recorded on a desktop computer running Spike 2 software (CED). Baseline neuronal activity was allowed to stabilise over a 30 minute period after which experimental protocols were performed.

Experimental protocols

- Characterization of putative visceral nociceptors

Flat-sheet preparations were assessed for mechanical sensitivity by gross mechanical probing with a blunt cotton bud, or longitudinal and/or circumferential stretch of the tissue (approximately 1cm performed manually using blunt forceps). Some preparations were tested for sensitivity to mucosal stroking with a metal rod following removal of respective pins from the anti-mesenteric border or tissue flanks; however, this was not systematically evaluated. The presence of nerve fibres responsive to tissue stretch was confirmed by repeat stretch (total 3x; 5 minute intervals) and the tissue re-pinned. In preparations responsive to focal mechanical probing by cotton bud, the receptive field was further defined using a 2g von Frey hair (Ugo Basile, Italy). Stimulus-response curves were generated by assessing the response to probing with a range of increasing strength von Frey hair probes (0.02, 0.04, 0.07, 0.16, 0.4, 1, 2 and 4 g; 3 x 3 second probe; at 3 second intervals). Two complete stimulus response curves were generated at 5 minute intervals. The acquisition of consistent probed responses was dependent on the location of the receptive field relative to the recording location within the mesentery and the topography of the tissue segment. Consequently, mechanosensitivity was not routinely studied in the mesentery as probing was likely to disrupt the nerve recording.

- Response of mechanosensitive fibres to algogenic mediators

In a subset of the preparations responsive to stretch or von Frey hair probing, the response of mechanosensitive units to the algogenic mediators bradykinin and/or ATP was assessed. Mediators (bradykinin 10µM to make an approximate bath concentration of 2 µM, or ATP 10mM to make an approximate bath concentration of 2mM) were superfused as a 20mL bolus in carbongenated Kreb's buffer into the tissue bath. Where both bradykinin and ATP

were applied sequentially, the response to ATP was investigated a minimum of 60 minutes following the application of bradykinin. A small number of initially mechanically insensitive preparations were probed with VFHs after the application of BK to investigate any changes in mechanosensitivity.

Effects of existing and novel therapeutic treatments for IBS on visceral nociceptor mechanosensitivity

- Controls

To determine the reproducibility and stability of repeated 2g VFH probing's, vehicle and time matched control experiments were performed. In these experiments, 20ml volumes of either DMSO 0.1% (n=2) or Krebs (n=3) were added following a baseline period of 3 sets of probing. Each set consisted of 3 probes applied for 3 seconds each and given at 3 second intervals. Sets of probes were applied every 5 minutes before and after vehicle, total test period 45 minutes.

- Role of $TRPV_4$ in human visceral nociceptor mechanosensitivity

The potential contribution of $TRPV_4$ to mechanosensitivity was assessed in a subset of preparations in which receptive fields sensitive to von Frey hair probing were identified. In these studies, the effects of bath superfusion (20ml bolus) with the selective $TRPV_4$ agonist GSK1016790A (10µM, to make an approximate bath concentration of 2µM), the selective $TRPV_4$ antagonist HC067047 (100µM, to make an approximate bath concentration of 20µM) were examined on repeat 0.4g von Frey hair probing (3 x 3 second probes at 3 second intervals). In each study 3 sets of repeated probes were performed prior to administration of GSK1016790A followed by 6-9 sets of probes, after which HC067047 was administered followed by 6-9 sets of probes. Each set of probes were applied at 5 minute intervals.

- Effect of 5-HT_4 partial agonist tegaserod on visceral nociceptor mechanosensitivity

The effect of tegaserod on mechanosensitivity was assessed in a subset of preparations with characterized visceral nociceptor units sensitive to von Frey hair probing. In these studies the effect of bath superfusion (100ml bolus) with tegaserod (30µM) was examined on repeated probing with 1g von Frey hair (3 x 3 second probes given at 3 second intervals, each set given 5 minutes apart) as described above.

Chemosensitivity of putative nociceptors

All preparations were tested for mechanosensitivity. A proportion of tissues either lacked measureable mechanical sensitivity or were unsuitable for repeated mechanical stimulation. These tissues were used exclusively for chemosensitivity protocols. In these studies drugs

were applied to the tissue bath by superfusion of a 20ml volume of: BK (10µM approximate bath concentration 2µM), ATP (10mM approximate bath concentration 2mM), 5-HT (1mM approximate bath concentration 200µM), histamine (3mM approximate bath concentration 600µM), PGE_2 (100µM approximate bath concentration 20µM), or capsaicin (10µM approximate bath concentration 2µM). Not all mediators were added to each preparation, but an effort was made to keep the mediator applications in the same order in each preparation. If a mediator failed to elicit a response, the next mediator was added 30 minutes later, otherwise a washout period of 60 minutes was observed.

- Mediator Pharmacology

For repeat application protocols, either 20ml of BK (10µM), ATP (10mM), histamine (3mM), 5-HT (1mM) were superfused into the bath 3 times consecutively, with a washout period of 60 minutes between applications. For pharmacological protocols involving BK and ATP, the first application of the mediator was superfused as normal. The preparation was then pre-treated by superfusion of an antagonist before the second application of the mediator. These were antagonists to the B1 receptor (R715, 300nM, 100ml), or the B2 receptor (HOE140, 300nM, 100ml or 1µM, 100ml) for BK or antagonists to the $P2X_{2/3}$, $_3$ receptors (RO4, 10µM, 100ml) or adenosine receptors (CGS 15943, 10µM, 100ml) for ATP. A second application of the appropriate mediator was superfused with the last 20ml of the antagonist. This was followed by a 60 minute washout period before the third application of the mediator was superfused into the bath. For pharmacological protocols involving repeat low concentrations of capsaicin (100nM given as a 1ml 10µM direct injection into the tissue bath), two applications were given with a 60 minute washout in between. After a 60 minute washout, a $TRPV_1$ antagonist (ABT-102, 2µM 100ml superfusion) was then given before the third application of capsaicin. Following a 60 minute washout, capsaicin was applied a fourth time.

The B1 receptor agonist Sar-[D-Phe^8]-des-Arg^9-Bradykinin was superfused into the bath (20ml, 100µM), to investigate its effect on afferent firing. To examine if activation of $P2X_3$ can stimulate HVAs, the $P2X_1$, $P2X_3$ agonist α, β methyleneadenosine trisphosphate (α, β meATP) was pipetted into the bath (1ml, 10mM).

Data analysis

Neuronal firing rates were examined offline using data analysis software in Spike 2 (CED, UK). For multi-unit recordings whole nerve activity was determined by counting the number of action potentials passing a threshold set at approximately twice the background noise level (~100µV). This activity was expressed as a rate histogram from which nerve activity could be measured. Electrical artefacts were removed from raw traces prior to figure

preparation. For few fibre recordings, individual units (typically 1-5) were discriminated using template matching software in Spike 2 to isolate individual waveforms as previously described[2] Single units were also discriminated from multi-unit recordings where spikes could be readily isolated due to differential amplitudes compared to other units. Once discriminated, activity was expressed as a rate histogram from which responses could be measured.

For protocols examining mechanosensitivity in serosal units the highest firing rate over two seconds was determined for each probe. From this each set of probes (at a given weight) was defined as the average of the highest two probes within the set. For interventional studies baseline was defined as the average of the three sets of probing prior to drug or vehicle administration and post intervention changes were determined from the average of each consecutive two sets of probing, e.g. average of probe set 4/5, set 5/6, set 6/7 etc. Spontaneous activity was examined in muscular and serosal units. For this the average firing rate over 100 seconds was determined from the rate histogram expressed in 1 second bins. For protocols examining chemosensitivity; positive responses to a given mediator were defined as a maintained increase (>25%) in action potential firing above baseline levels [3]. Responses to a mediator were determined from the rate histogram expressed in 20 second bins. Baseline were calculated by averaging the firing rate over a 100 second period prior to mediator application and the change in firing rate post mediator calculated by subtracting baseline firing from the peak response within 10 minutes of mediator application.

Changes in response proportions between two groups were statistically compared using Fisher's exact test and the effect size was statistically compared using paired or unpaired Student's t-tests as appropriate. Comparisons between multiple groups were performed using a one way ANOVA with Bonferroni post hoc analysis between groups. Finally, correlations were performed between variables using Pearson or Spearman tests as appropriate. Significance was set at $p<0.05$.

Post Hoc Analysis

Post hoc analysis were performed to examine the effect of cold storage, age, gender and region of tissue studied, on mechanosensitivity (VFH probing) and chemosensitivity (BK and ATP), in macroscopically normal tissue from surgical resections performed for the treatment of bowel cancer. Additionally, the effects of inflammatory disease were examined by comparing responses with those obtained from inflamed tissue obtained from resections performed for the surgical treatment of inflammatory bowel disease (IBD); Crohn's disease and ulcerative colitis.

Mouse Electrophysiology

A 3cm section of the descending colon was placed in a bespoke tissue bath (10ml volume), cannulated at both ends, and luminally perfused (0.1ml/min) with carbongenated (95% O_2, 5% CO_2) Kreb's buffer (in mM: 124.0 NaCl, 4.8 KCl, 1.3 NaH_2PO_4, 1.2 $MgSO_4.7H_20$, 2.5 $CaCl_2$, 11.1 Glucose, 25.0 $NaHCO_3$) supplemented with nifedipine (10µM) and atropine (10µM) to block the contraction of smooth muscle, and indomethacin (3µM) to inhibit endogenous prostanoid production. The tissue was also serosally perfused (7ml/min; 32-34°C) with supplemented carbongenated Krebs buffer. Lumbar colonic nerve bundles were carefully dissected free and ongoing nerve discharge was recorded using a borosilicate glass pipette suction electrode with reference to a common silver wire reference/ground electrode using a differential amplifier (gain 5K) and headstage (Neurolog Ltd, UK). The resultant signal was band passed (100-1500Hz; Neurolog, UK) and digitally filtered (50 Hz; Humbug, Quest Scientific, UK), before being digitised (20kHz, 1401 micro Cambridge Electronic Design; CED, UK) and recorded on a desktop computer running Spike 2 software (CED).

Following a minimum 20 minute stabilisation period, rapid phasic ramp distensions (0–80 mm Hg, 60 seconds at 9 min intervals) were performed by switching luminal perfusion out-flow of the cannulated colon to an 80 mm Hg end pressure. After 3 comparable baseline responses had been attained, responses to phasic distensions was investigated in the presence of sequential bath superfusion of the $TRPV_4$ antagonist HC067047 (50ml) at increasing concentrations (1, 20 and 100µM, in 1% DMSO/supplemented carbongenated Krebs).

SUPPLEMENTARY METHODS REFERENCES

1 De Fontgalland D, Wattchow DA, Costa M, Brookes SJ. Immunohistochemical characterization of the innervation of human colonic mesenteric and submucosal blood vessels. *Neurogastroenterol Motil* 2008;**20**:1212-26.

2 Hillsley K, Kirkup AJ, Grundy D. Direct and indirect actions of 5-hydroxytryptamine on the discharge of mesenteric afferent fibres innervating the rat jejunum. *J Physiol* 1998;**506 (Pt 2)**:551-61.

3 Hicks GA, Coldwell JR, Schindler M, Ward PA, Jenkins D, Lynn PA*, et al.* Excitation of rat colonic afferent fibres by 5-HT(3) receptors. *J Physiol* 2002;**544**:861-9.

Supplementary Table 1

Pt. #	Age	Sex	Tissue Region	Disease	Cold Storage
1	30	F	Ileum	Crohn’s Disease	No
2	49	F	Sigmoid Colon	Diverticular Disease	No
3	52	M	Sigmoid Colon	Cancer	No
4	50	F	Sigmoid Colon	Cancer	No
5	71	M	Sigmoid Colon	Collapsed Stoma	No
6	80	F	Caecum	Reformation of Stoma	Yes
7	41	F	Ileum	Crohn’s Disease Stricture	Yes
8	65	M	Asc. Colon	Polyps	No
9	20	M	Asc. Colon	Ulcerative Colitis	No
10	69	F	Sigmoid Colon	Cancer	No
11	60	M	Sigmoid Colon	Cancer	Yes
12	78	M	Sigmoid Colon	Cancer	No
13	64	M	Sigmoid Colon	Cancer	Yes
14	78	M	Sigmoid Colon	Cancer	Yes
15	76	F	Ileum	Cancer	No
16	47	M	Sigmoid Colon	Polyps	Yes
17	60	M	Ileum	Trauma	No
18	76	F	Sigmoid Colon	Cancer	Yes
19	58	F	Ileum	Adhesions	No
20	33	M	Ileum	Crohn’s Disease	No
21	63	F	Colon	Cancer	No
22	65	M	Trans. Colon	PSC	No
23	65	F	Sigmoid Colon	Cancer	No
24	24	F	Ileum	Crohn’s Disease	No

**25**	**57**	**F**	**Sigmoid Colon**	**Cancer**	**No**
**26**	**61**	**F**	**Trans. Colon**	**Diverticular Disease**	**Yes**
**27**	**84**	**M**	**Trans. Colon**	**Cancer**	**No**
**28**	**20**	**M**	**Rectum**	**Crohn’s Disease**	**No**
**29**	**73**	**M**	**Sigmoid Colon**	**Cancer**	**No**
**30**	**37**	**F**	**Rectum**	**Ulcerative Colitis**	**No**
**31**	**39**	**M**	**Sigmoid Colon**	**Cancer**	**No**
**32**	**78**	**M**	**Desc. Colon**	**Cancer**	**No**
**33**	**78**	**M**	**Desc. Colon**	**Cancer**	**Yes**
**34**	**62**	**F**	**Appendix**	**Cancer**	**No**
**35**	**27**	**M**	**Appendix**	**Ulcerative Colitis**	**No**
**36**	**27**	**M**	**Sigmoid Colon**	**Ulcerative Colitis**	**Yes**
**37**	**52**	**M**	**Sigmoid Colon**	**Cancer**	**No**
**38**	**68**	**M**	**Rectum**	**Cancer**	**No**
**39**	**72**	**F**	**Appendix**	**Cancer**	**No**
**40**	**72**	**F**	**Asc. Colon**	**Cancer**	**Yes**
**41**	**52**	**F**	**Rectum**	**Cancer**	**No**
**42**	**39**	**F**	**Ileum**	**Crohn's Disease**	**No**
**43**	**54**	**M**	**Sigmoid Colon**	**Cancer**	**No**
**44**	**73**	**M**	**Sigmoid Colon**	**Cancer**	**No**
**45**	**54**	**F**	**Sigmoid Colon**	**Polyps**	**No**
**46**	**84**	**F**	**Appendix**	**Cancer**	**No**
**47**	**45**	**F**	**Rectum**	**Cancer**	**No**
**48**	**64**	**M**	**Sigmoid Colon**	**Cancer**	**Yes**
**49**	**66**	**M**	**Sigmoid Colon**	**Cancer**	**No**
**50**	**60**	**F**	**Sigmoid Colon**	**Cancer**	**Yes**
**51**	**82**	**M**	**Asc. Colon**	**Cancer**	**No**

52	48	F	Caecum	Cancer	No
53	16	M	Ileum	Crohn's Disease	Yes
54	61	M	Sigmoid Colon	Cancer	No
55	54	F	Rectum	Cancer	No
56	72	F	Rectum	Cancer	No
57	85	M	Rectum	Cancer	No
58	72	M	Appendix	Cancer	No
59	72	M	Sigmoid Colon	Cancer	Yes
60	51	F	Sigmoid Colon	Diverticular Disease	Yes
61	60	M	Sigmoid Colon	Diverticular Disease	Yes
62	26	M	Appendix	Appendicitis	Yes
63	55	M	Appendix	Ulcerative Colitis	No
64	24	M	Asc. Colon	Cancer	Yes
65	44	F	Sigmoid Colon	Persistent Prolapse	Yes
66	22	M	Sigmoid Colon	Ulcerative Colitis	No
67	64	M	Sigmoid Colon	Cancer	No
68	60	F	Desc. Colon	Attenuated FAP	Yes
69	85	M	Sigmoid Colon	Cancer	Yes
70	48	F	Sigmoid Colon	Diverticular Disease	No
71	68	F	Sigmoid Colon	Diverticular Disease	Yes
72	71	M	Sigmoid Colon	Cancer	No
73	64	F	Ileum	Cancer	Yes
74	54	M	Sigmoid Colon	Cancer	Yes
75	53	F	Sigmoid Colon	Cancer	No
76	47	M	Rectum	Ulcerative Colitis	No
77	45	M	Sigmoid Colon	Diverticular Disease	Yes
78	47	M	Sigmoid Colon	Diverticular Disease	No

79	19	M	Appendix	Appendicitis	Yes
80	63	F	Sigmoid Colon	Cancer	No
81	60	M	Asc. Colon	Cancer	Yes
82	71	F	Sigmoid Colon	Cancer	Yes
83	87	M	Sigmoid Colon	Cancer	Yes
84	41	M	Sigmoid Colon	Cancer	Yes
85	48	M	Sigmoid Colon	Cancer	Yes
86	87	M	Asc. Colon	Cancer	No
87	85	M	Sigmoid Colon	Cancer	Yes
88	18	M	Ileum	Crohn’s Disease	No
89	64	F	Sigmoid Colon	Stricture	Yes
90	87	M	Rectum	Cancer	No
91	50	F	Sigmoid Colon	Cancer	No
92	50	F	Sigmoid Colon	Cancer	Yes
93	56	M	Sigmoid Colon	Chronic Constipation	Yes
94	64	M	Rectum	Cancer	Yes
95	55	M	Desc. Colon	Cancer	Yes
96	62	M	Sigmoid Colon	Cancer	Yes
97	74	M	Desc. Colon	Cancer	Yes

Supplementary Figure 2

Pt. #	Age	Sex	Tissue Region	Disease	Cold Storage
2	49	F	Sigmoid Colon	Diverticular Disease	NO
3	52	M	Sigmoid Colon	Cancer	No
4	50	F	Sigmoid Colon	Cancer	No
11	60	M	Sigmoid Colon	Cancer	Yes
13	78	M	Sigmoid Colon	Cancer	Yes
19	58	F	Ileum	Adhesions	No
28	20	M	Rectum	Crohn's Disease	No
29	73	M	Sigmoid Colon	Cancer	No
32	78	M	Des. Colon	Cancer	No
47	45	F	Rectum	Cancer	No
50	60	F	Sigmoid Colon	Cancer	Yes
51	82	M	Asc. Colon	Cancer	No
54	61	M	Sigmoid Colon	Cancer	No
55	54	F	Rectum	Cancer	No
56	72	F	Rectum	Cancer	No
59	72	M	Sigmoid Colon	Cancer	Yes
60	51	F	Sigmoid Colon	Diverticular Disease	Yes
72	71	M	Sigmoid Colon	Cancer	No
74	54	M	Sigmoid Colon	Cancer	Yes
89	64	F	Sigmoid Colon	Stricture	Yes
91	50	F	Sigmoid Colon	Cancer	No
94	64	M	Rectum	Cancer	Yes

**96**	**62**	**M**	**Sigmoid Colon**	**Cancer**	**Yes**

Supplementary Table 3

Pt. #	Age	Sex	Tissue Region	Disease	Cold Storage
10	69	F	Sigmoid Colon	Cancer	No
12	78	M	Sigmoid Colon	Cancer	No
13	64	M	Sigmoid Colon	Cancer	Yes
15	76	F	Ileum	Cancer	No
16	47	M	Sigmoid Colon	Polyps	Yes
19	58	F	Ileum	Adhesions	No
26	61	F	Trans. Colon	Diverticular Disease	Yes
28	20	M	Rectum	Crohn’s Disease	No
29	73	M	Sigmoid Colon	Cancer	No
31	39	M	Sigmoid Colon	Cancer	No
32	78	M	Desc. Colon	Cancer	No
51	82	M	Asc. Colon	Cancer	No
66	22	M	Sigmoid Colon	Ulcerative Colitis	No
67	64	M	Sigmoid Colon	Cancer	No
69	85	M	Sigmoid Colon	Cancer	Yes
70	48	F	Sigmoid Colon	Diverticular Disease	No
72	71	M	Sigmoid Colon	Cancer	No
73	64	F	Ileum	Cancer	Yes

Supplementary Table 4

Pt. #	Age	Sex	Tissue Region	Disease	Cold Storage
7	41	F	Ileum	Crohn's Disease Stricture	Yes
11	60	M	Sigmoid Colon	Cancer	Yes

Supplementary Table 5

Pt. #	Age	Sex	Tissue Region	Disease	Cold Storage
2	49	F	Sigmoid Colon	Diverticular Disease	No
3	52	M	Sigmoid Colon	Cancer	No
89	64	F	Sigmoid Colon	Stricture	Yes
91	50	F	Sigmoid Colon	Cancer	No
97	74	M	Des. Colon	Cancer	Yes

Supplementary Table 6

Pt. #	Age	Sex	Tissue Region	Disease	Cold Storage
10	69	F	Sigmoid Colon	Cancer	No
19	58	F	Ileum	Adhesions	No
26	61	F	Trans. Colon	Diverticular Disease	Yes
28	20	M	Rectum	Crohn's Disease	No
29	73	M	Sigmoid Colon	Cancer	No
47	45	F	Rectum	Cancer	No

Supplementary Table 7

Pt. #	Age	Sex	Tissue Region	Disease	Cold Storage
3	52	M	Sigmoid Colon	Cancer	No
4	50	F	Sigmoid Colon	Cancer	No
32	78	M	Desc. Colon	Cancer	No

Supplementary Table 8

Pt. #	Age	Sex	Tissue Region	Disease	Cold Storage
10	69	F	Sigmoid Colon	Cancer	No
19	58	F	Ileum	Adhesions	No
26	61	F	Trans. Colon	Diverticular Disease	Yes
28	20	M	Rectum	Crohn’s Disease	No
32	78	M	Des. Colon	Cancer	No
47	45	F	Rectum	Cancer	No
74	54	M	Sigmoid Colon	Cancer	Yes

Supplementary Table 9

Pt. #	Age	Sex	Tissue Region	Disease	Cold Storage
54	61	M	Sigmoid Colon	Cancer	No
55	54	F	Rectum	Cancer	No
56	72	F	Rectum	Cancer	No
72	71	M	Sigmoid Colon	Cancer	No
74	54	M	Sigmoid Colon	Cancer	Yes
77	45	M	Sigmoid Colon	Diverticular Disease	Yes

Supplementary Table 10

Pt. #	Age	Sex	Tissue Region	Disease	Cold Storage
5	71	M	Sigmoid Colon	Collapsed Stoma	No
6	80	F	Caecum	Reformation of Stoma	Yes
8	65	M	Asc. Colon	Polyps	No
9	20	M	Asc. Colon	Ulcerative Colitis	No
16	47	M	Sigmoid Colon	Polyps	Yes
17	60	M	Ileum	Trauma	No
18	76	F	Sigmoid Colon	Cancer	Yes
20	33	M	Ileum	Crohn’s Disease	No
21	63	F	Colon	Cancer	No
22	65	M	Trans. Colon	PSC	No
23	65	F	Sigmoid Colon	Cancer	No
25	57	F	Sigmoid Colon	Cancer	No
26	61	F	Trans. Colon	Diverticular Disease	Yes
27	84	M	Trans. Colon	Cancer	No
30	37	F	Rectum	Ulcerative Colitis	No
33	78	M	Desc. Colon	Cancer	Yes
36	27	M	Sigmoid Colon	Ulcerative Colitis	Yes
37	52	M	Sigmoid Colon	Cancer	No
38	68	M	Rectum	Cancer	No
40	72	F	Asc. Colon	Cancer	Yes
41	52	F	Rectum	Cancer	No
42	39	F	Ileum	Crohn's Disease	No
43	54	M	Sigmoid Colon	Cancer	No
44	73	M	Sigmoid Colon	Cancer	No
45	54	F	Sigmoid Colon	Multiple Polyps	No
48	64	M	Sigmoid Colon	Cancer	Yes
49	66	M	Sigmoid Colon	Cancer	No

**52**	**48**	**F**	**Ceacum**	**Cancer**	**No**
**53**	**16**	**M**	**Ileum**	**Crohn's Disease**	**Yes**
**57**	**85**	**M**	**Rectum**	**Cancer**	**No**
**61**	**60**	**M**	**Sigmoid Colon**	**Diverticular Disease**	**Yes**
**64**	**24**	**M**	**Asc. Colon**	**Cancer**	**Yes**
**65**	**44**	**F**	**Sigmoid Colon**	**Persistent Prolapse**	**Yes**
**68**	**60**	**F**	**Desc. Colon**	**Attenuated FAP**	**Yes**
**71**	**68**	**F**	**Sigmoid Colon**	**Diverticular Disease**	**Yes**
**74**	**54**	**M**	**Sigmoid Colon**	**Cancer**	**Yes**
**75**	**53**	**F**	**Sigmoid Colon**	**Cancer**	**No**
**76**	**47**	**M**	**Rectum**	**Ulcerative Colitis**	**No**
**77**	**45**	**M**	**Sigmoid Colon**	**Diverticular Disease**	**Yes**
**78**	**47**	**M**	**Sigmoid Colon**	**Diverticular Disease**	**No**
**80**	**63**	**F**	**Sigmoid Colon**	**Cancer**	**No**
**81**	**60**	**M**	**Asc. Colon**	**Cancer**	**Yes**
**82**	**71**	**F**	**Sigmoid Colon**	**Cancer**	**Yes**
**83**	**87**	**M**	**Sigmoid Colon**	**Cancer**	**Yes**
**84**	**41**	**M**	**Sigmoid Colon**	**Cancer**	**Yes**
**85**	**48**	**M**	**Sigmoid Colon**	**Cancer**	**Yes**
**86**	**87**	**M**	**Asc. Colon**	**Cancer**	**No**
**87**	**85**	**M**	**Sigmoid Colon**	**Cancer**	**Yes**
**88**	**18**	**M**	**Ileum**	**Crohn’s Disease**	**No**
**90**	**87**	**M**	**Rectum**	**Cancer**	**No**
**92**	**50**	**F**	**Sigmoid Colon**	**Cancer**	**Yes**

Supplementary Table 11

Pt. #	Age	Sex	Tissue Region	Disease	Cold Storage
19	58	F	Ileum	Adhesions	No
22	65	M	Trans. Colon	PSC	No
24	24	F	Ileum	Crohn's Disease	No
25	57	F	Sigmoid Colon	Cancer	No
79	19	M	Appendix	Appendicitis	Yes
80	63	F	Sigmoid Colon	Cancer	No

Supplementary Table 12

Pt. #	Age	Sex	Tissue Region	Disease	Cold Storage
36	27	M	Sigmoid Colon	Ulcerative Colitis	Yes
39	72	F	Appendix	Cancer	No
42	39	F	Ileum	Crohn's Disease	No
43	54	M	Sigmoid Colon	Cancer	No

Supplementary Table 13

Pt. #	Age	Sex	Tissue Region	Disease	Cold Storage
71	68	F	Sigmoid Colon	Diverticular Disease	Yes
79	19	M	Appendix	Appendicitis	Yes

Supplementary Table 14

Pt. #	Age	Sex	Tissue Region	Disease	Cold Storage
22	65	M	Trans. Colon	PSC	No
64	24	M	Asc. Colon	Cancer	Yes

Supplementary Table 15

Pt. #	Age	Sex	Tissue Region	Disease	Cold Storage
32	78	M	Desc. Colon	Cancer	No
34	62	F	Appendix	Cancer	No
35	27	M	Appendix	Ulcerative Colitis	No
37	52	M	Sigmoid Colon	Cancer	No
38	68	M	Rectum	Cancer	No
39	72	F	Appendix	Cancer	No

Supplementary Table 16

Pt. #	Age	Sex	Tissue Region	Disease	Cold Storage
74	54	M	Sigmoid Colon	Cancer	Yes
81	60	M	Asc. Colon	Cancer	Yes
83	87	M	Sigmoid Colon	Cancer	Yes
85	48	M	Sigmoid Colon	Cancer	Yes

Supplementary Table 17

Pt. #	Age	Sex	Tissue Region	Disease	Cold Storage
46	84	F	Appendix	Cancer	No
48	64	M	Sigmoid Colon	Cancer	Yes
51	82	M	Ascending Colon	Cancer	No
57	85	M	Rectum	Cancer	No
58	72	M	Appendix	Cancer	No
61	60	M	Sigmoid Colon	Diverticular Disease	Yes

Supplementary Table 18

Pt. #	Age	Sex	Tissue Region	Disease	Cold Storage
62	26	M	Appendix	Appendicitis	Yes
63	55	M	Appendix	Ulcerative Colitis	No
65	44	F	Sigmoid Colon	Persistent Prolapse	Yes
69	85	M	Sigmoid Colon	Cancer	Yes
70	48	F	Sigmoid Colon	Diverticular Disease	No
71	68	F	Sigmoid Colon	Diverticular Disease	Yes

Supplementary Table 19

Pt. #	Age	Sex	Tissue Region	Disease	Cold Storage
93	56	M	Sigmoid Colon	Chronic Constipation	Yes
94	64	M	Rectum	Cancer	Yes
95	55	M	Descending Colon	Cancer	Yes

Supplementary Table 20

Pt. #	Age	Sex	Tissue Region	Disease	Cold Storage
3	52	M	Sigmoid Colon	Cancer	No
4	50	F	Sigmoid Colon	Cancer	No
10	69	F	Sigmoid Colon	Cancer	No
11	60	M	Sigmoid Colon	Cancer	Yes
12	78	M	Sigmoid Colon	Cancer	No
13	64	M	Sigmoid Colon	Cancer	Yes
18	76	F	Sigmoid Colon	Cancer	Yes
23	65	F	Sigmoid Colon	Cancer	No
25	57	F	Sigmoid Colon	Cancer	No
27	84	M	Trans. Colon	Cancer	No
29	73	M	Sigmoid Colon	Cancer	No
31	39	M	Sigmoid Colon	Cancer	No
32	78	M	Desc. Colon	Cancer	No
34	62	F	Appendix	Cancer	No
37	52	M	Sigmoid Colon	Cancer	No
38	68	M	Rectum	Cancer	No
39	72	F	Appendix	Cancer	No
40	72	F	Asc. Colon	Cancer	Yes
41	52	F	Rectum	Cancer	No
43	54	M	Sigmoid Colon	Cancer	No
44	73	M	Sigmoid Colon	Cancer	No
46	84	F	Appendix	Cancer	No
47	45	F	Rectum	Cancer	No

48	64	M	Sigmoid Colon	Cancer	Yes
49	66	M	Sigmoid Colon	Cancer	No
51	82	M	Asc. Colon	Cancer	No
54	61	M	Sigmoid Colon	Cancer	No
55	54	F	Rectum	Cancer	No
56	72	F	Rectum	Cancer	No
57	85	M	Rectum	Cancer	No
58	72	M	Appendix	Cancer	No
59	72	M	Sigmoid Colon	Cancer	Yes
64	24	M	Asc. Colon	Cancer	Yes
67	64	M	Sigmoid Colon	Cancer	No
69	85	M	Sigmoid Colon	Cancer	Yes
72	71	M	Sigmoid Colon	Cancer	No
74	54	M	Sigmoid Colon	Cancer	Yes
75	53	F	Sigmoid Colon	Cancer	No
80	63	F	Sigmoid Colon	Cancer	No
81	60	M	Asc. Colon	Cancer	Yes
82	71	F	Sigmoid Colon	Cancer	Yes
83	87	M	Sigmoid Colon	Cancer	Yes
84	41	M	Sigmoid Colon	Cancer	Yes
85	48	M	Sigmoid Colon	Cancer	Yes
91	50	F	Sigmoid Colon	Cancer	No
92	50	F	Sigmoid Colon	Cancer	Yes
94	64	M	Rectum	Cancer	Yes
95	55	M	Desc. Colon	Cancer	Yes
96	62	M	Sigmoid Colon	Cancer	Yes

Supplementary Table 21

Pt. #	Age	Sex	Tissue Region	Disease	Cold Storage
3	52	M	Sigmoid Colon	Cancer	No
4	50	F	Sigmoid Colon	Cancer	No
10	69	F	Sigmoid Colon	Cancer	No
11	60	M	Sigmoid Colon	Cancer	Yes
12	78	M	Sigmoid Colon	Cancer	No
13	64	M	Sigmoid Colon	Cancer	Yes
18	76	F	Sigmoid Colon	Cancer	Yes
23	65	F	Sigmoid Colon	Cancer	No
64	24	M	Asc. Colon	Cancer	Yes
25	57	F	Sigmoid Colon	Cancer	No
27	84	M	Trans. Colon	Cancer	No
29	73	M	Sigmoid Colon	Cancer	No
31	39	M	Sigmoid Colon	Cancer	No
32	78	M	Desc. Colon	Cancer	No
34	62	F	Appendix	Cancer	No
37	52	M	Sigmoid Colon	Cancer	No
38	68	M	Rectum	Cancer	No
39	72	F	Appendix	Cancer	No
40	72	F	Asc. Colon	Cancer	Yes
41	52	F	Rectum	Cancer	No
43	54	M	Sigmoid Colon	Cancer	No
44	73	M	Sigmoid Colon	Cancer	No
46	84	F	Appendix	Cancer	No
47	45	F	Rectum	Cancer	No

48	64	M	Sigmoid Colon	Cancer	Yes
49	66	M	Sigmoid Colon	Cancer	No
51	82	M	Asc. Colon	Cancer	No
54	61	M	Sigmoid Colon	Cancer	No
55	54	F	Rectum	Cancer	No
56	72	F	Rectum	Cancer	No
57	85	M	Rectum	Cancer	No
58	72	M	Appendix	Cancer	No
59	72	M	Sigmoid Colon	Cancer	Yes
67	64	M	Sigmoid Colon	Cancer	No
69	85	M	Sigmoid Colon	Cancer	Yes
72	71	M	Sigmoid Colon	Cancer	No
74	54	M	Sigmoid Colon	Cancer	Yes
75	53	F	Sigmoid Colon	Cancer	No
80	63	F	Sigmoid Colon	Cancer	No
81	60	M	Asc. Colon	Cancer	Yes
82	71	F	Sigmoid Colon	Cancer	Yes
83	87	M	Sigmoid Colon	Cancer	Yes
84	41	M	Sigmoid Colon	Cancer	Yes
85	48	M	Sigmoid Colon	Cancer	Yes
90	87	M	Rectum	Cancer	No
91	50	F	Sigmoid Colon	Cancer	No
92	50	F	Sigmoid Colon	Cancer	Yes
94	64	M	Rectum	Cancer	Yes
95	55	M	Desc. Colon	Cancer	Yes
96	62	M	Sigmoid Colon	Cancer	Yes

Supplementary Table 22

Pt. #	Age	Sex	Tissue Region	Disease	Cold Storage
1	30	F	Ileum	Crohn’s Disease	No
3	52	M	Sigmoid Colon	Cancer	No
4	50	F	Sigmoid Colon	Cancer	No
7	41	F	Ileum	Crohn’s Disease Stricture	Yes
9	20	M	Asc. Colon	Ulcerative Colitis	No
10	69	F	Sigmoid Colon	Cancer	No
11	60	M	Sigmoid Colon	Cancer	Yes
12	78	M	Sigmoid Colon	Cancer	No
13	64	M	Sigmoid Colon	Cancer	Yes
18	76	F	Sigmoid Colon	Cancer	Yes
23	65	F	Sigmoid Colon	Cancer	No
24	24	F	Ileum	Crohn’s Disease	No
25	57	F	Sigmoid Colon	Cancer	No
27	84	M	Trans. Colon	Cancer	No
28	20	M	Rectum	Crohn’s	No
29	73	M	Sigmoid Colon	Cancer	No
30	37	F	Rectum	Ulcerative Colitis	No
31	39	M	Sigmoid Colon	Cancer	No
32	78	M	Desc. Colon	Cancer	No
34	62	F	Appendix	Cancer	No
36	27	M	Sigmoid Colon	Ulcerative Colitis	Yes
37	52	M	Sigmoid Colon	Cancer	No
38	68	M	Rectum	Cancer	No
39	72	F	Appendix	Cancer	No

40	72	F	Asc. Colon	Cancer	Yes
41	52	F	Rectum	Cancer	No
42	39	F	Ileum	Crohn's Disease	No
43	54	M	Sigmoid Colon	Cancer	No
44	73	M	Sigmoid Colon	Cancer	No
46	84	F	Appendix	Cancer	No
47	45	F	Rectum	Cancer	No
48	64	M	Sigmoid Colon	Cancer	Yes
49	66	M	Sigmoid Colon	Cancer	No
51	82	M	Asc. Colon	Cancer	No
54	61	M	Sigmoid Colon	Cancer	No
55	54	F	Rectum	Cancer	No
56	72	F	Rectum	Cancer	No
57	85	M	Rectum	Cancer	No
58	72	M	Appendix	Cancer	No
59	72	M	Sigmoid Colon	Cancer	Yes
64	24	M	Asc. Colon	Cancer	Yes
67	64	M	Sigmoid Colon	Cancer	No
69	85	M	Sigmoid Colon	Cancer	Yes
72	71	M	Sigmoid Colon	Cancer	No
74	54	M	Sigmoid Colon	Cancer	Yes
75	53	F	Sigmoid Colon	Cancer	No
76	47	M	Rectum	Ulcerative Colitis	No
80	63	F	Sigmoid Colon	Cancer	No
81	60	M	Asc. Colon	Cancer	Yes
82	71	F	Sigmoid Colon	Cancer	Yes

**83**	**87**	**M**	**Sigmoid Colon**	**Cancer**	**Yes**
**84**	**41**	**M**	**Sigmoid Colon**	**Cancer**	**Yes**
**85**	**48**	**M**	**Sigmoid Colon**	**Cancer**	**Yes**
**88**	**18**	**M**	**Ileum**	**Crohn’s Disease**	**No**
**91**	**50**	**F**	**Sigmoid Colon**	**Cancer**	**No**
**92**	**50**	**F**	**Sigmoid Colon**	**Cancer**	**Yes**
**94**	**64**	**M**	**Rectum**	**Cancer**	**Yes**
**95**	**55**	**M**	**Desc. Colon**	**Cancer**	**Yes**
**96**	**62**	**M**	**Sigmoid Colon**	**Cancer**	**Yes**

SUPPLEMENTARY FIGURE/TABLE LEGENDS

Supplementary Figure 1

Application of the $TRPV_4$ agonist GSK1016790A directly activates a proportion of afferents innervating the human intestine. (A) Rate histogram and neurogram showing the direct activation of a human afferent fibre by GSK1016790A. (B) Bar graph demonstrating the significant increase in firing rate (n=3, p<0.05), and (C) pie chart illustrating the proportion of responding preparations (3/14 preparations tested) to GSK1016790A. Mean ± SEM. *p<0.05, paired t-test.

Supplementary Figure 2

The $TRPV_4$ antagonist HC067047 inhibits mouse splanchnic afferent response to repeated phasic distensions. (A) Mean frequency firing rate graph and pressure plot showing a reduction in the splanchnic afferent activity in response to repeated colonic phasic distensions (80mmHg), following application of 1µM and 20µM HC067047. (B) Bar graph demonstrating response inhibition at 20µM HC067047 (n=3, p<0.05). These experiments were used to determine the optimal dose for use in human studies. Mean ± SEM. *p<0.05, one way ANOVA.

Supplementary Figure 3

The B1 receptor agonist Sar-[D-Phe^8]-des-Arg^9-Bradykinin failed to activate afferent fibres. The P2X agonist α, β methylene ATP activates afferents innervating the human intestine. Bar graphs showing the firing rate before and after (A) Sar-[D-Phe^8]-des-Arg^9-Bradykinin (n=14, p>0.05) or (B) α, β methylene ATP (n=1) application. (C) Neurogram demonstrating the activation of human afferents by α, β methylene ATP, and (D) rate histogram and single unit analysis of the neurogram showing the effect α, β methylene ATP has on a single afferent fibre. Mean ± SEM. NS = Not significant, *p*>0.05, paired t-test.

Supplementary Figure 4

Responses to low doses of capsaicin are sensitive to $TRPV_1$ blockade. Rate histogram and neurogram illustrating a human colonic afferent response to repeated application of capsaicin, its inhibition by co-application of the $TRPV_1$ antagonist ABT-102, and wash out of the antagonist response in a responsive single unit.

Supplementary Table 1

Table of information for every patient included in the publication including assigned patient number, age at operation, tissue region, sex, disease, and whether the tissue was cold

stored overnight before use. Asc. = Ascending. Desc. = Descending. Trans = Transverse. PSC= Primary sclerosing cholangitis. FAP = Familial adenomatous polyposis.

Supplementary Table 2

Table of information for each patient in which serosal afferents were identified including assigned patient number, age at operation, tissue region, sex, disease, and whether the tissue was cold stored overnight before use. Asc. = Ascending. Desc. = Descending.

Supplementary Table 3

Table of information for each patient in which muscular afferents were identified including assigned patient number, age at operation, tissue region, sex, disease, and whether the tissue was cold stored overnight before use. Asc. = Ascending. Desc. = Descending. Trans = Transverse.

Supplementary Table 4

Table of information for each patient in which silent afferents were identified including assigned patient number, age at operation, tissue region, sex, disease, and whether the tissue was cold stored overnight before use.

Supplementary Table 5

Table of information for each patient whose tissue was used for vehicle (Krebs/DMSO) von Frey hair probing time matched control experiments including assigned patient number, age at operation, tissue region, sex, disease, and whether the tissue was cold stored overnight before use. Desc. = Descending.

Supplementary Table 6

Table of information for each patient whose tissue was used for $TRPV_4$ agonist GSK1016790A von Frey hair probing experiments including assigned patient number, age at operation, tissue region, sex, disease, and whether the tissue was cold stored overnight before use. Trans = Transverse.

Supplementary Table 7

Table of information for each patient whose tissue directly responded to the application of the $TRPV_4$ agonist GSK1016790A including assigned patient number, age at operation, tissue region, sex, disease, and whether the tissue was cold stored overnight before use. Desc. = Descending.

Supplementary Table 8

Table of information for each patient whose tissue was used for $TRPV_4$ antagonist HC067047 von Frey hair probing experiments including assigned patient number, age at operation, tissue region, sex, disease, and whether the tissue was cold stored overnight before use. Trans = Transverse. Desc. = Descending.

Supplementary Table 9

Table of information for each patient whose tissue was used for tegaserod von Frey hair probing experiments including assigned patient number, age at operation, tissue region, sex, disease, and whether the tissue was cold stored overnight before use. Trans = Transverse.

Supplementary Table 10

Table of information for each patient whose tissue was used for repeat bradykinin experiments including assigned patient number, age at operation, tissue region, sex, disease, and whether the tissue was cold stored overnight before use. Trans = Transverse. PSC= Primary sclerosing cholangitis.

Supplementary Table 11

Table of information for each patient whose tissue was used for repeat ATP experiments including assigned patient number, age at operation, tissue region, sex, disease, and whether the tissue was cold stored overnight before use.

Supplementary Table 12

Table of information for each patient whose tissue was used for repeat histamine experiments including assigned patient number, age at operation, tissue region, sex, disease, and whether the tissue was cold stored overnight before use.

Supplementary Table 13

Table of information for each patient whose tissue was used for repeat 5-HT experiments including assigned patient number, age at operation, tissue region, sex, disease, and whether the tissue was cold stored overnight before use. Trans = Transverse. Desc. = Descending . PSC= Primary sclerosing cholangitis.

Supplementary Table 14

Table of information for each patient whose tissue was used for B1 receptor antagonist R715 experiments including assigned patient number, age at operation, tissue region, sex, disease, and whether the tissue was cold stored overnight before use.

Supplementary Table 15

Table of information for each patient whose tissue was used for B2 receptor antagonist HOE140 (300nM) experiments including assigned patient number, age at operation, tissue region, sex, disease, and whether the tissue was cold stored overnight before use. Desc. = Descending .

Supplementary Table 16

Table of information for each patient whose tissue was used for B2 receptor antagonist HOE140 (1µM) experiments including assigned patient number, age at operation, tissue region, sex, disease, and whether the tissue was cold stored overnight before use. Asc. = Ascending.

Supplementary Table 17

Table of information for each patient whose tissue was used for P1 receptor antagonist CGS15943 experiments including assigned patient number, age at operation, tissue region, sex, disease, and whether the tissue was cold stored overnight before use.

Supplementary Table 18

Table of information for each patient whose tissue was used for P2X receptor antagonist RO4 experiments including assigned patient number, age at operation, tissue region, sex, disease, and whether the tissue was cold stored overnight before use.

Supplementary Table 19

Table of information for each patient whose tissue was used for chemosensitivity experiments including assigned patient number, age at operation, tissue region, sex, disease, and whether the tissue was cold stored overnight before use. Asc. = Ascending. Desc. = Descending. Trans = Transverse. PSC= Primary sclerosing cholangitis. FAP = Familial adenomatous polyposis.

Supplementary Table 20

Table of information for each patient whose tissue was used for gender post hoc analysis including assigned patient number, age at operation, tissue region, sex, disease, and

whether the tissue was cold stored overnight before use. Asc. = Ascending. Desc. = Descending. Trans = Transverse.

Supplementary Table 21

Table of information for each patient whose tissue was used for age and cold storage post hoc analysis including assigned patient number, age at operation, tissue region, sex, disease, and whether the tissue was cold stored overnight before use. Asc. = Ascending. Desc. = Descending. Trans = Transverse.

Supplementary Table 22

Table of information for each patient whose tissue was used for disease post hoc analysis including assigned patient number, age at operation, tissue region, sex, disease, and whether the tissue was cold stored overnight before use. Asc. = Ascending. Desc. = Descending. Trans = Transverse.

Supplementary Figures

1-4

Supplementary Figure 1

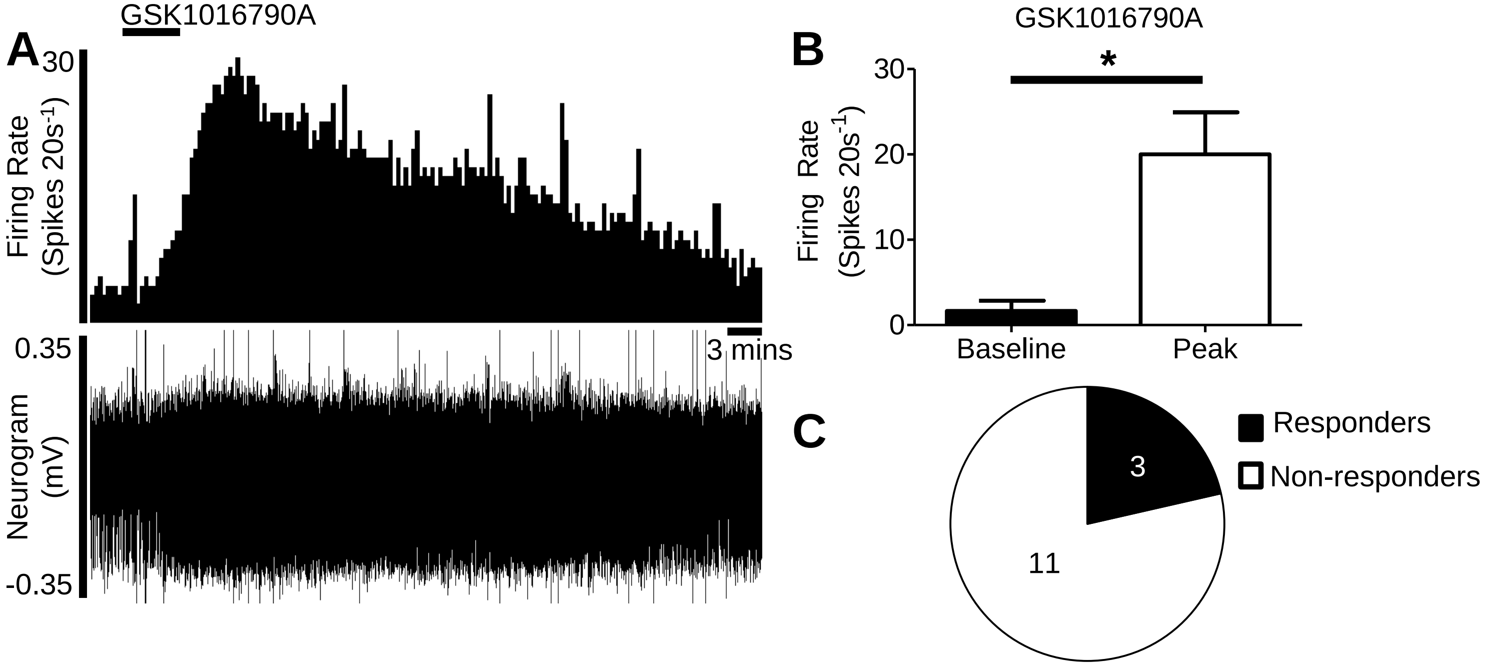

Supplementary Figure 2

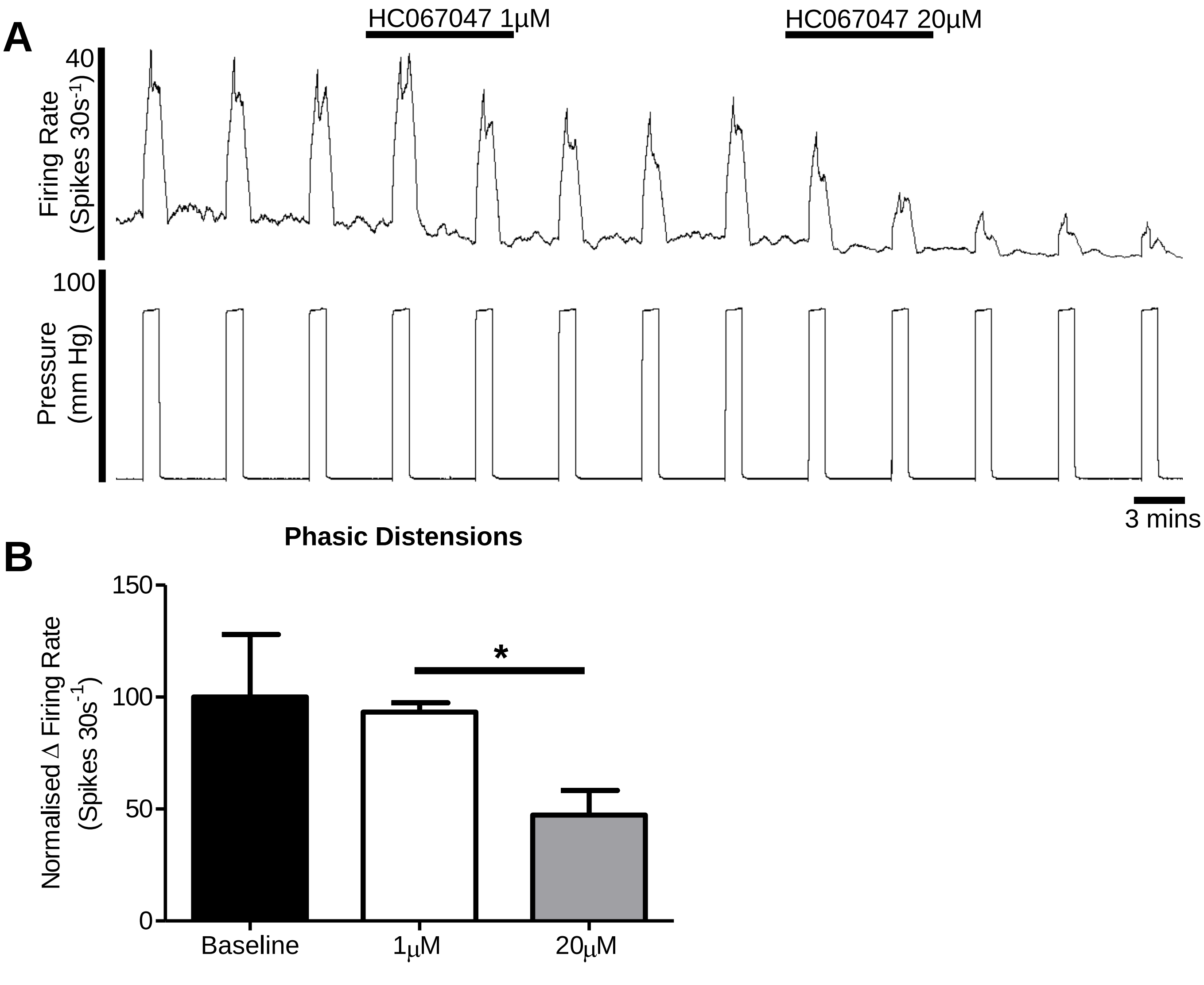

Supplementary Figure 3

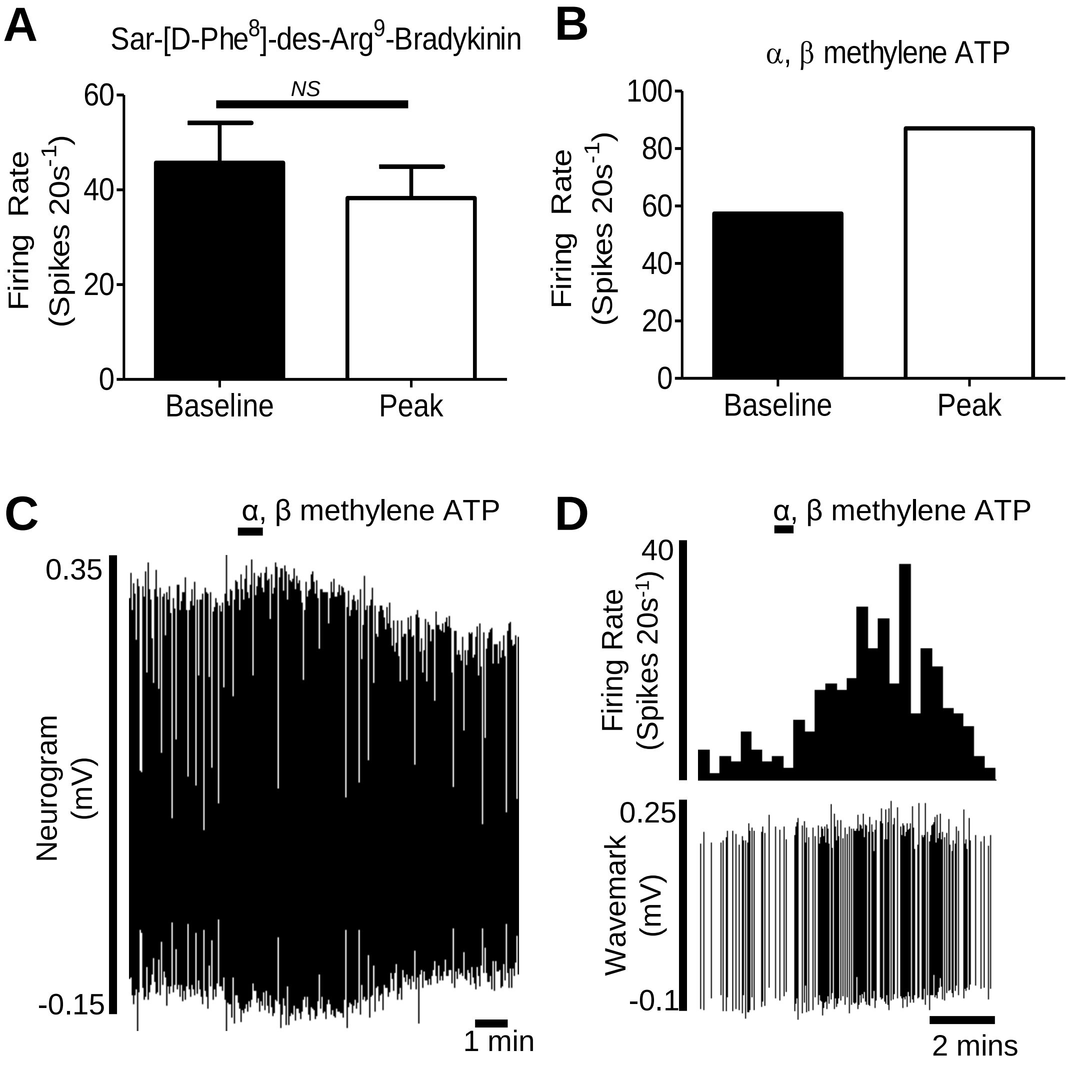

Supplementary Figure 4

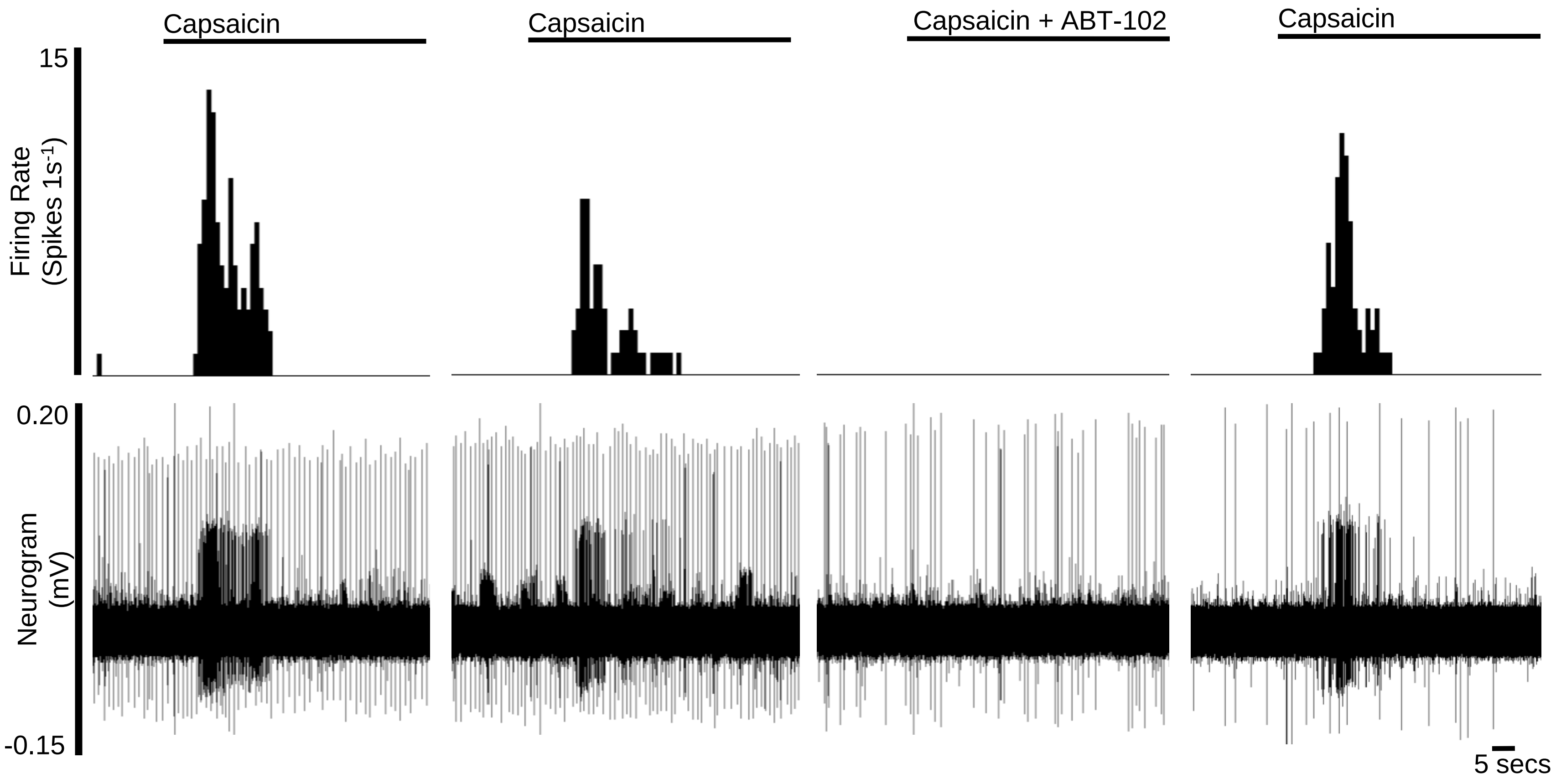

Supplement: Supplementary data [file gutjnl-2016-311629supp001.pdf]
